# Scalable CAR-T production in a 2-litre perfusion stirred-tank bioreactor with automated harvesting and scale-down model characterisation

**DOI:** 10.3389/fbioe.2025.1694134

**Published:** 2026-01-12

**Authors:** Pierre Springuel, Pedro Silva Couto, Dale J. Stibbs, Michal Szelwicki, Amanda Frangleton, Timo Schmidberger, Ajith George, Fern Slingsby, Nicola Bevan, Asma Ahmad, Rachel Legmann, Noushin Dianat, Rukmini Ladi, Julia Hengst, Qasim A. Rafiq

**Affiliations:** 1 Department of Biochemical Engineering, University College London, London, United Kingdom; 2 Sartorius Stedim UK Limited, Surrey, United Kingdom; 3 Sartorius Stedim Biotech GmbH, Goettingen, Germany; 4 Essen BioScience Ltd. (Part of the Sartorius Group), Royston, United Kingdom; 5 Repligen Corporation, Waltham, MA, United States; 6 Sartorius Stedim France S.A.S., Aubagne, France; 7 Sartorius Stedim North America Inc., Bohemia, NY, United States

**Keywords:** CAR-T cell, scale-up, stirred-tank bioreactor, perfusion, scale-down model, automated downstream processing

## Abstract

The emergence of allogeneic, universal chimeric antigen receptor (CAR) T cell therapies requires intensified and scalable manufacturing workflows supported by representative scale-down models (SDMs) to enable efficient process development and future large-scale production of off-the-shelf therapies. Here, we present a 7-day CAR-T cell expansion process intensified via perfusion of serum-free medium in a 2 L Univessel® Single-Use stirred-tank bioreactor (STR), consistently achieving 30 × 10^6^ cells/mL, corresponding to 113 ± 7 anti-CD19 CAR-T doses per batch. Parallel runs in 250 mL Ambr® 250 STRs conducted at equivalent volumetric power input (*P/V*) of ∼8.78 W/m^3^ demonstrated comparable process performance and final product quality, with univariate and multivariate analyses of cell growth, phenotype, cytotoxicity, and cytokine secretion validating the Ambr® 250 as a predictive SDM for the 2 L process. Integrating capacitance sensing in the 2 L STR enabled robust monitoring of viable cell concentrations in real-time, with strong correlation to offline measurements (R^2^ = 0.98). For downstream processing, the Ksep® 400 was used to automate CAR-T cell harvesting, concentration, and washing at the 2 L scale, achieving >90% product recovery and nine-fold volume reduction without impacting product quality attributes compared to manual methods. This study establishes a scalable CAR-T manufacturing workflow supported by a predictive SDM, providing an efficient platform for process development and scale-up to enable future large-scale production of allogeneic CAR-T cell therapies.

## 1 Introduction

The substantial therapeutic efficacy of autologous chimeric antigen receptor (CAR) T cell therapies has had a transformative impact on the treatment of hematologic malignancies leading to the approval of seven CAR T cell products since 2017 (Patel et al., 2025). However, the complex nature of autologous CAR T cell manufacturing continues to significantly limit the accessibility, affordability, and commercial viability of these therapies (Shah et al., 2023). The multi-step process involving the collection of T cells from an individual patient via apheresis, followed by their genetic modification and expansion *ex vivo*, and subsequent transport back to clinical sites for reinfusion, poses substantial logistical and manufacturing challenges associated with patient-derived starting material which is often of sub-optimal quality (Dias et al., 2024). These unique constraints limit production capacity and result in extended manufacturing timelines of 3–6 weeks elevating costs per CAR-T dose to $400–500K (Agliardi et al., 2025). There is therefore a need for more streamlined and scalable manufacturing approaches that can reduce costs and improve patient access.

The development of allogeneic, universal CAR-T therapies offers a promising avenue to overcome the unique challenges associated with autologous manufacturing (Depil et al., 2020; Diorio et al., 2024). By genetically engineering healthy donor-derived T cells to express the therapeutic CAR receptor and evade immune rejection through the knockout of genes such as the T-cell receptor (TCR) and human leukocyte antigen (HLA), CAR-T products could be manufactured at large-scale in advance, eliminating long production lead times and enabling immediate treatment with therapies available off-the-shelf that can be administered to patients universally (Chen and van den Brink, 2024).

Investigational allogeneic anti-CD19 CAR-T cell therapies have shown early promise in the treatment of B-cell haematological malignancies, with Phase 1 trials reporting favourable safety profiles and overall complete response rates comparable with those reported for approved autologous products (Ghobadi et al., 2024; Mansoori et al., 2024; Locke et al., 2025). With Phase II trials now underway (WU-CART-007, NCT06514794), (ALLO-501A, NCT06500273), manufacturing science and technologies must keep pace with clinical progress to enable the large-scale production of allogeneic CAR-T therapies and support future commercial translation. Achieving widely accessible, off-the-shelf CAR-T therapies at reduced costs per dose will require scalable upstream and downstream manufacturing processes capable of producing large numbers of high-quality doses at economies of scale.

Stirred-tank bioreactors (STRs) have been widely used for decades in the large-scale production of biologics and have significantly contributed to the democratisation of blockbuster drugs such as monoclonal antibodies and vaccines (Sharma et al., 2022). While the scalability of manufacturing platforms designed for single-dose autologous CAR-T products such as the CliniMACS Prodigy are inherently constrained (Garcia-Aponte et al., 2021), STRs offer the advantages of proven scalability beyond 10,000 L, operational flexibility and extensive bioprocessing and regulatory prior knowledge in the production of proteins, viral vectors and increasingly, cellular therapies (Nienow, 2014; Silva Couto et al., 2020; Ganeeva et al., 2022) While the expansion of CAR-T cells has been demonstrated in 0.015–0.25 L STRs (Costariol et al., 2020; Lamas et al., 2022; Hood et al., 2025; Silva Couto et al., 2025; Springuel et al., 2025), demonstrations at larger scales (0.5-1 L) have been limited to non-modified T-cell cultures (Ou et al., 2019; Gatla et al., 2022; Selvarajan et al., 2024). Similarly, scalable downstream solutions that can enable automated bioreactor harvesting and final CAR-T product concentration have only been assessed with non-modified T-cells (Gatla et al., 2022). Characterising the impact of scalable, multi-litre upstream and downstream manufacturing workflows on CAR-T cell critical quality attributes, such as CAR transgene expression and cytotoxicity is therefore still required. Additionally, the integration of process analytical technologies (PATs) that can enable real-time process monitoring of critical parameters such as CAR-T cell concentrations *in situ* is yet to be demonstrated.

To further support large-scale CAR-T process development, there also remains a need to validate representative scale-down models (SDMs) that can accurately mimic CAR-T bioreactor processes at the manufacturing scale. Widely used in commercial biologics manufacturing, SDMs are essential tools that enable high-throughput, small-scale process development at lower costs. They allow therapy developers to systematically screen and optimise critical process parameters and assess their impact on product quality attributes in accordance with Quality by Design (QbD) principles (Sandner et al., 2018; Manahan et al., 2019; Deshmukh and Ogunyankin, 2020). Given the lack of validated SDMs for CAR-T manufacturing and the impracticality of extensive process development at large scale, the validation of robust and predictive SDMs will be key to accelerating the development of scalable allogeneic CAR-T manufacturing workflows.

To address these needs, this study characterises a CAR-T cell expansion process intensified via perfusion of serum-free medium in a 2 L single-use STR and evaluates critical quality attributes, including CAR-T cell growth, phenotype, cytotoxicity, and cytokine secretion. Process scalability was assessed by performing the same process in parallel in a 250 mL STR using matched volumetric power input (P/V) and process performance was compared to evaluate the 250 mL system as a representative SDM. Additionally, the integration of capacitance sensing via dielectric spectroscopy was investigated at the 2 L scale for real-time monitoring of CAR-T cell concentrations. The study also includes the integration and characterisation of the Ksep® 400 at the 2 L scale for the automation of bioreactor harvesting, CAR-T product concentration and washing steps.

## 2 Results

### 2.1 Comparable CAR-T cell expansion in 250 mL and 2 L bioreactors

**FIGURE 1:**
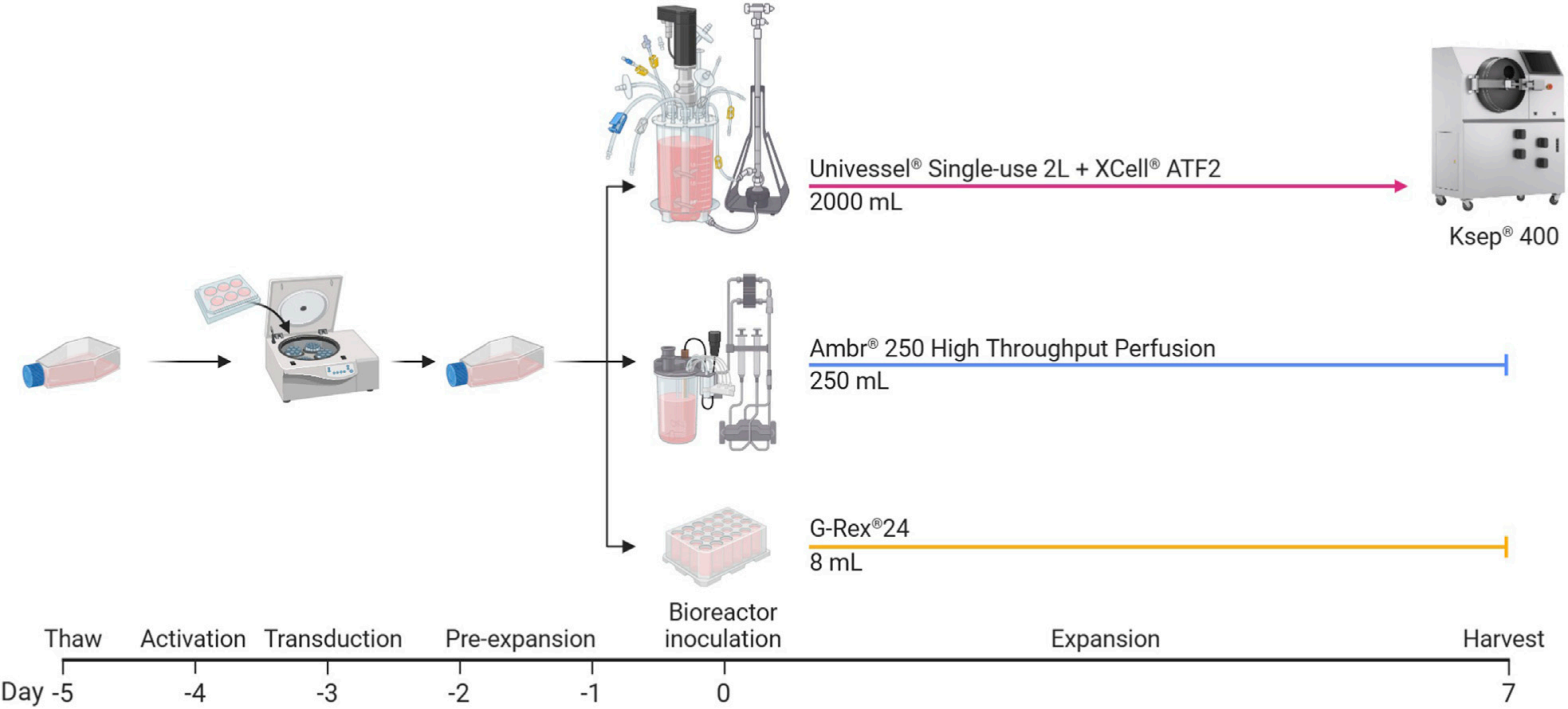
Overview of CAR-T cell expansion experiments in 250 mL vs. 2 L perfusion stirred-tank bioreactors and static well-plates. One day post-lentiviral transduction via spinoculation, anti-CD19 CAR-T cells were pre-expanded for 2 days in static T-flasks. On Day 0, CAR-T cells were inoculated into the Ambr^®^ 250 and Univessel^®^ 2 L stirred-tank bioreactors (STR) and G-Rex®24 well-plates in parallel and expanded for 7 days. STR working volumes were increased by 110% on Day 1 and ATF perfusion was initiated on Day 2 at 1.0 vessel volume exchanges per day until the end of experiments. On Day 7, the Ksep® 400 was used to automated cell harvesting, washing and concentration from the 2 L STR. In well-plates, a 75% medium exchange was performed on Day 4. All experiments were performed in triplicate (n = 3) using cells from a single healthy donor.

To assess the scalability and reproducibility of a previously optimised perfusion CAR-T cell expansion process, anti-CD19 CAR-T cells derived from a single healthy donor were cultured in parallel in 250 mL (Ambr® 250 High-Throughput Perfusion) and 2 L (Univessel® Single-Use) STRs. Cultures were maintained for 7 days in serum-free medium, with the initiation of alternating tangential flow (ATF) perfusion on Day 2 at a fixed rate of 1.0 vessel volume exchanges per day (VVD). The agitation rate in the 2 L STR was scaled to maintain an equivalent P/V as in the 250 mL STR process, calculated as ∼8.78 W/m^3^ using Equation 1. STR perfusion processes were benchmarked against G-Rex® 24 gas-permeable well-plate cultures, which served as static controls (Figure 1).

**FIGURE 2:**
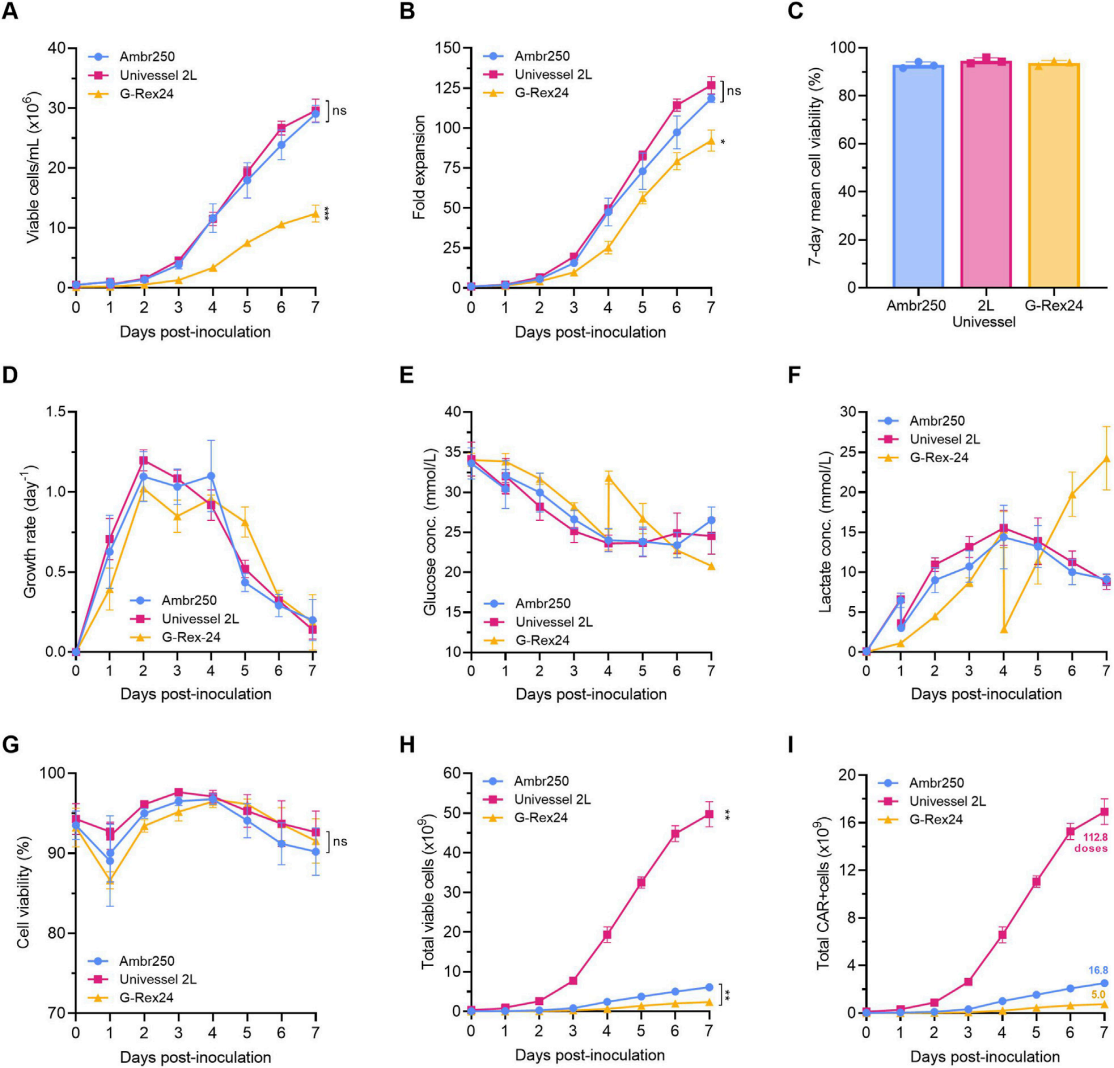
Comparable CAR-T cell growth in 250 mL and 2 L perfusion bioreactors. Overview of **(A)** daily viable cell densities and **(B)** fold expansions. **(C)** Seven-day mean cell viability and **(D)** daily cellular growth rates. **(E)** Daily glucose and **(F)** lactate bioreactor concentrations. **(G)** Daily cell viabilities, **(H)** total viable cell yields and **(I)** total CAR + cell yields with the total number of representative CAR-T doses produced per batch indicated, calculated by multiplying the Day 7 total cell yields by the respective Day 7 CD3^+^ CAR transduction efficiencies shown in Figure 5B and assuming one dose as 150 × 10^6^ CAR+ cells. G-Rex®24 well-plate dose yields were calculated assuming 24 wells filled with 8 mL. Data presented as mean ± SD of n = 3 experimental replicates using one healthy donor with well-plate data presented as the mean of n = 3 technical replicate wells per experimental replicate (n = 9). Statistical analyses were performed on Day 7 values using Welch’s ANOVA and Dunnett’s T3 multiple comparison test with *p < 0.05, **p < 0.01, and ***p < 0.001 on Day 7 values, ns = non-significant.

Cellular growth profiles were highly comparable between the 250 mL and 2 L STR perfusion processes (Figure 2). Cultures were initiated at 0.5 × 10^6^ cells/mL and reached comparable final cell densities of 29.0 ± 1.5 × 10^6^ cells/mL and 29.6 ± 1.9 × 10^6^ cells/mL (Figure 2A), corresponding to final fold expansions of 118.4 ± 2.4 and 126.8 ± 5.4, in the 250 mL and 2 L bioreactors, respectively (Figure 2B). These data solely reflect CAR-T cell growth in the STRs from Days 0–7 and exclude cell growth observed during the activation, transduction and pre-expansion steps in T-flasks (Days −5 to 0). Combining the cell growth achieved over the entirety of the 12-day process resulted in total mean fold expansions of 409–437 in STRs (Supplementary Figure S1). Cell viability trends were also consistent across both STR scales, with average viabilities exceeding 90% throughout the culture period and remaining comparable despite the use of different ATF perfusion equipment at the 250 mL (syringe pumps) and 2 L scale (diaphragm pump) (Figure 2C). Together, these findings indicated a high degree of similarity between 250 mL and 2 L STR perfusion processes based on matched P/V and suggested that neither bioreactor scale nor perfusion mechanism impacted CAR-T cell growth nor viability. The lack of significant differences in cell viabilities between STR processes and static well plate controls further emphasised good CAR-T cell tolerability to any shear stresses imparted by the ATF perfusion process or agitation via impeller-based stirring in the STRs.

### 2.2 Perfusion in stirred-tank bioreactors supports superior CAR-T cell expansion

Implementation of perfusion to enable the continuous exchange of culture medium significantly enhanced CAR-T cell expansion relative to static well plate cultures in which a single 75% medium exchange was performed on Day 4 (Figures 2A,B). After adjustment for different inoculation cell concentrations (0.5 × 10^6^ cells/mL in STRs vs. 0.125 × 10^6^ cells/mL in well plates), perfusion STR cultures achieved 34% greater fold expansions compared to well plates (∼123 vs. ∼90-fold; p < 0.05) (Figure 2B). This superior cell growth was correlated with higher trending growth rates in the STRs early in the process from Days 0–4 (Figure 2D). The superior growth rates in STRs were likely driven by a combination of continuous nutrient supply via perfusion, enhanced mixing and gas transfer via impeller-based agitation and the control of culture pH and dissolved oxygen (DO), all of which were absent in static well-plates cultures.

Analysis of daily glucose and lactate concentrations confirmed that superior cell growth in STRs was supported by a more stable metabolic environment. The continuous perfusion of fresh culture medium stabilised glucose levels, which plateaued at approximately 24 mmol/L by Day 4 in STR cultures but dropped below this threshold by Day 6 in well plate cultures (Figure 2E). In well plates, lactate levels progressively accumulated to ∼24 mmol/L by Day 7, whereas in perfusion STRs they peaked at 15 mmol/L by Day 4 before being cleared by perfusion and progressively dropping to ∼9 mmol/L by the end of cultures (Figure 2F). Metabolic profiles were very comparable between the 250 mL and 2 L STR cultures, with glucose depletion and lactate accumulation peaking by Day 4 and subsequently reversing (Figures 2E,F), and with glucose consumption and lactate production rate trends similarly decreasing over time (Supplementary Figure S2). Notably, despite a fixed perfusion rate during continued cell expansion, the reversal in glucose depletion and lactate accumulation after Day 4 suggested that medium supply exceeded metabolic demand. This indicated that late-stage perfusion rates could potentially be reduced in future processes to conserve medium and reduce costs.

Post-inoculation, cell viabilities dropped by 2%–7% across all bioreactor systems during the first day of culture, before recovering to 93%–96% by Day 2 (Figure 2G). Given that this decline and recovery was also observed in static well plates, the potential negative impact of impeller-driven agitation in the STRs was considered an unlikely cause, particularly since STR viabilities were higher than those in well plates from Days 0–4. Instead, we hypothesised that the early drop in viability reflected cellular adaptation to pH shifts when transferring cells from more acidic pre-expansion flask cultures where lactate accumulation from proliferation between Days −2 and 0 had lowered the pH, into the more basic fresh medium of the bioreactors. However, the absence of pH and metabolite measurements during the pre-expansion phase prevented confirmation of this hypothesis.

A gradual decline in cell viabilities beginning on Day 3 was observed in the 2 L STR and on Day 4 in the 250 mL STR (Figure 2G). The onset of this decline coincided with lactate concentrations surpassing 10 mmol/L and peaking near 15 mmol/L (Figure 2F), ranges which have been reported as toxic thresholds for T cell cultures (Quinn et al., 2020; Uhl et al., 2020). To assess whether cell viabilities would recover, cultures in the 250 mL bioreactor were extended to 10 days which revealed eventual stabilisation of cell viabilities at 87% by Days 9–10 (Supplementary Figure S3a). Separately, this confirmed sustained CAR-T cell expansion beyond Day 7 to final concentrations of ∼47 × 10^6^ cells/mL (Supplementary Figure S3b), indicating that extending culture duration could have further improved overall yields in the 2 L STR cultures.

Further comparison of the CAR-T cell perfusion process with a preliminary fed-batch process using non-transduced T cells (n = 1) highlighted the benefits of implementing perfusion (Supplementary Figure S4). The perfusion process yielded a 3.4-fold increase in final cell concentrations relative to fed-batch in both 250 mL and 2 L scales and supported superior growth rates throughout the cultures (Supplementary Figures S4a–c). Notably, viabilities in fed-batch cultures began to decline from Day 2, correlating with lactate levels exceeding 10–15 mmol/L and thus further supporting the hypothesis of a lactate-related toxic threshold around these concentrations (Supplementary Figures S4d–f). It is important to note that the use of non-transduced T cells in the fed-batch process represents a confounding factor as cellular metabolism and activation states likely differed between non-transduced and CAR-T cells. Consequently, this was not a direct comparison, and these findings should be considered as indicative rather than fully comparable. We have previously compared fed-batch and perfusion modes more rigorously using CAR-T cells exclusively in the 250 mL STR system, where similar improvements in final cell yields of 3–4.5-fold were observed (Hood et al., 2025; Springuel et al., 2025). Together, these results support the role of perfusion to intensify CAR-T cell expansion to maximise final cell yields.

### 2.3 2 L perfusion scale-up yields 100+ CAR-T doses per batch

Scale-up of the perfusion process to the 2 L STR achieved final cell yields of 49.7 ± 3.2 × 10^9^ total viable cells by Day 7 (Figure 2H). Based on a transduction efficiency of 34% at harvest at this scale (Figure 5B), this corresponded to 16.9 ± 1.1 × 10^9^ total CAR-T cells (Figure 2I). Assuming a therapeutic CAR-T dose of 150 × 10^6^ CAR + cells (Benjamin et al., 2022; Locke et al., 2025), the 2 L STR expansion process therefore yielded 112.8 ± 7 CAR-T doses per batch, demonstrating the significant number of doses achievable by process scale-up. The number of doses in the 250 mL STR expansion process was calculated similarly using the respective CAR transduction achieved at this scale, resulting in 16.8 ± 0.8 doses per batch. To account for the sacrificial sampling that was performed in the G-Rex24 well-plates over the 7-day cultures, theoretical total cell and dose yields were calculated assuming 24 wells filled with 8 mL working volume each, resulting in a total of 5.1 ± 0.6 doses per plate within 7 days. For reference, each well plate was inoculated with a total of 24 × 10^6^ cells compared with 50 × 10^6^ cells in the 250 mL STR. To account for this difference and enable a comparison with the 250 mL STR process, normalising to an equivalent inoculum increased the theoretical well-plate yields to 10.6 doses. Table 1 summarises the total cellular and dose yields achieved by each process.

**TABLE 1:** Summary of bioreactor process cellular and CAR-T dose yields (n = 3).

Parameter (Day 7)	Ambr® 250	Univessel® 2 L	G-Rex®24
Working volume (mL)	210	1,680	24 × 8 = 192
Fold expansion	118.4 ± 2.4	126.8 ± 5.4	89.6 ± 7.1
Total viable cells (x10^9^)	6.1 ± 0.3	49.7 ± 3.2	2.4 ± 0.3
%CAR+ of CD3+	41.4 ± 2.4	34.1 ± 3.2	32.0 ± 1.3
Total CAR-T cells (x10^9^)	2.5 ± 0.1	16.9 ± 1.1	0.8 ± 0.08
Total CAR-T doses	16.8 ± 0.8	112.8 ± 7.2	5.0 ± 0.6

### 2.4 Comparable environmental control between 250 mL and 2 L bioreactors

To ensure comparable mixing intensity and shear conditions across scales, the impeller agitation rate at the 2 L scale was scaled to match the P/V in the 250 mL STR. To assess whether P/V-based scaling enabled comparable oxygen transfer, dissolved oxygen (DO) levels were monitored in both bioreactor scales.

**FIGURE 3:**
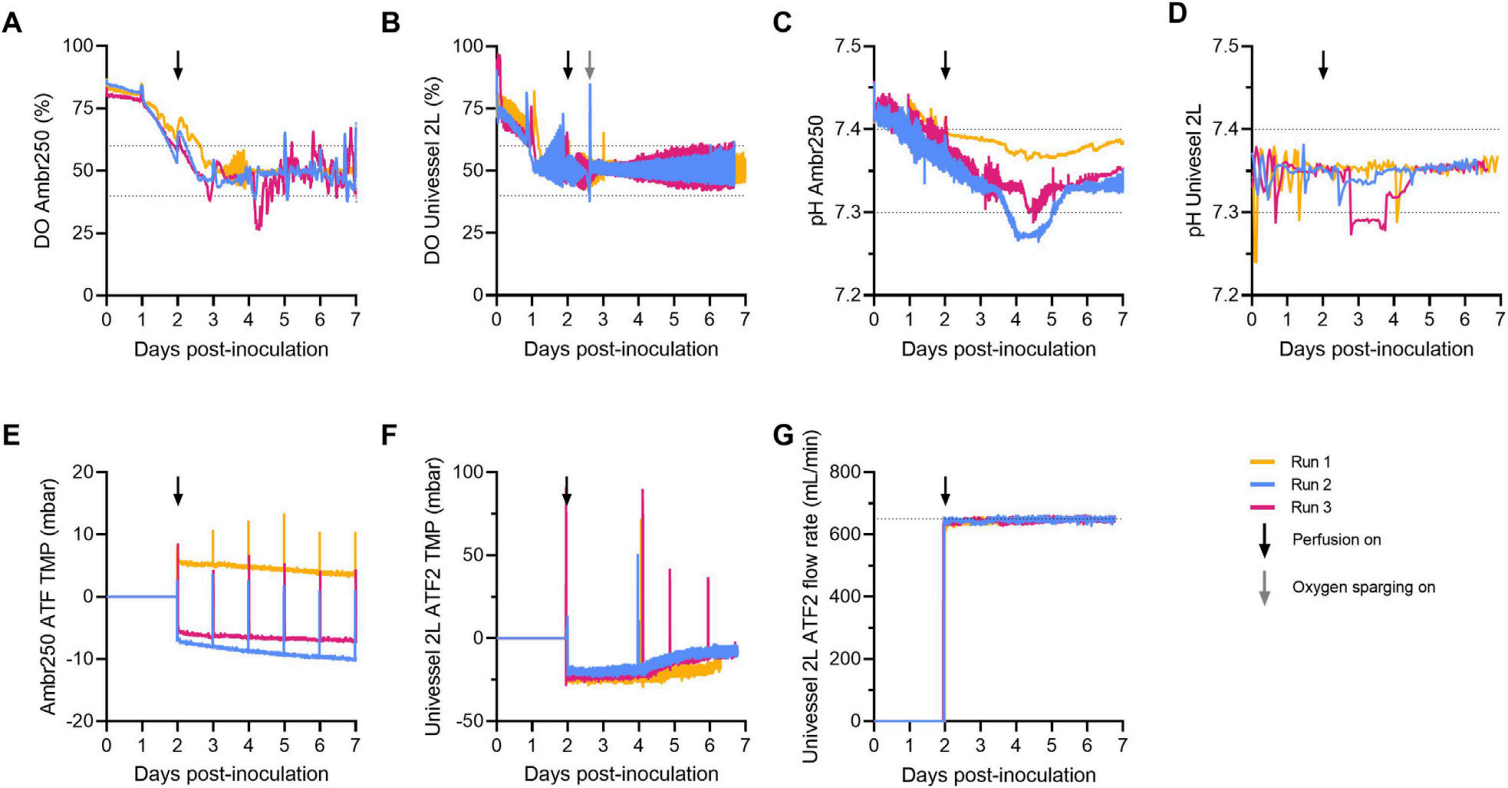
Comparable pH and dissolved oxygen trends in 250 mL and 2 L perfusion bioreactors. **(A)** Spot-probe on-line monitoring of dissolved oxygen (DO) in the Ambr^®^ 250 and **(B)** Univessel^®^ 2 L stirred-tank bioreactors (STRs). **(C)** Spot-probe on-line monitoring of pH in the Ambr^®^ 250 and **(D)** Univessel^®^ 2 L STRs. **(E)** Integrated Ambr^®^ 250 hollow fiber perfusion filter transmembrane pressure (TMP), **(F)** 2 L STR XCell® ATF2 perfusion device TMP and **(G)** alternating tangential flow rate. Data presented for n = 3 experimental replicates. Horizontal dotted lines indicate parameter setpoint deadbands.

In both STR processes, DO levels were allowed to naturally decline to 50%, after which they were maintained at this setpoint using headspace gassing of nitrogen and oxygen at vessel volume–normalised flow rates (Figures 3A,B). However, preliminary 2 L experiments revealed that headspace gassing alone was insufficient to sustain the 50% DO setpoint beyond Day 3, indicating a limitation in oxygen transfer (Supplementary Figure S5). This highlighted that P/V-based agitation scaling did not achieve fully equivalent gas–liquid mass transfer compared to the 250 mL process, likely due to subtle differences in headspace geometries between the two bioreactor scales. To address this, pure oxygen sparging was introduced as required from Day 3 onwards in the 2 L bioreactor, supplementing headspace gassing and successfully maintaining DO at the target setpoint (Figure 3B). Despite the difference in oxygen control strategy, DO levels were maintained within 50% ± 10% in both STR scales, with sparging at 2 L enabling tighter control than headspace gassing alone at the 250 mL scale. Importantly, CAR-T cells tolerated any additional shear stress from sparging, as evidenced by comparable cell viabilities between the two scales (Figure 2C).

In 250 mL and 2 L STRs, pH was successfully maintained at the target of 7.35 ± 0.05 via bolus base additions and headspace gassing of carbon dioxide (Figures 3C,D). Brief dips below 7.25 occurred in two runs between Days 3 and 5 but were promptly corrected by the bioreactor pH control system. The overall pH trends declined from inoculation through to Day 4, followed by a recovery phase which correlated with the trends in lactate accumulation and subsequent clearance (Figure 2F).

To investigate potential fouling of the 0.2 µm hollow-fiber perfusion filters, transmembrane pressure (TMP) was monitored throughout the culture period. While transient TMP spikes were observed during temporary pausing of perfusion to allow the replacement of fresh medium and waste bottles, no sustained pressure increases occurred at either bioreactor scale, indicating minimal filter fouling (Figures 3E,F). At the 2 L scale, the XCell® ATF2 perfusion flow rate was operated and well controlled at 650 mL/min (Figure 3G), to match the estimated shear rates of ∼1,500 s^-1^ imparted by the Ambr® 250 perfusion system using Equation 6. Comparable mean cell viabilities between 250 mL and 2 L processes supported the conclusion that similar shear conditions were achieved in both perfusion processes (Figure 2C). Separately, analysis of cell-free permeate fractions confirmed the absence of cells in the permeate, verifying effective cell retention and concentration within the bioreactors (data not shown).

### 2.5 Multifrequency capacitance measurement enables real-time monitoring of CAR-T cell concentrations

**FIGURE 4:**
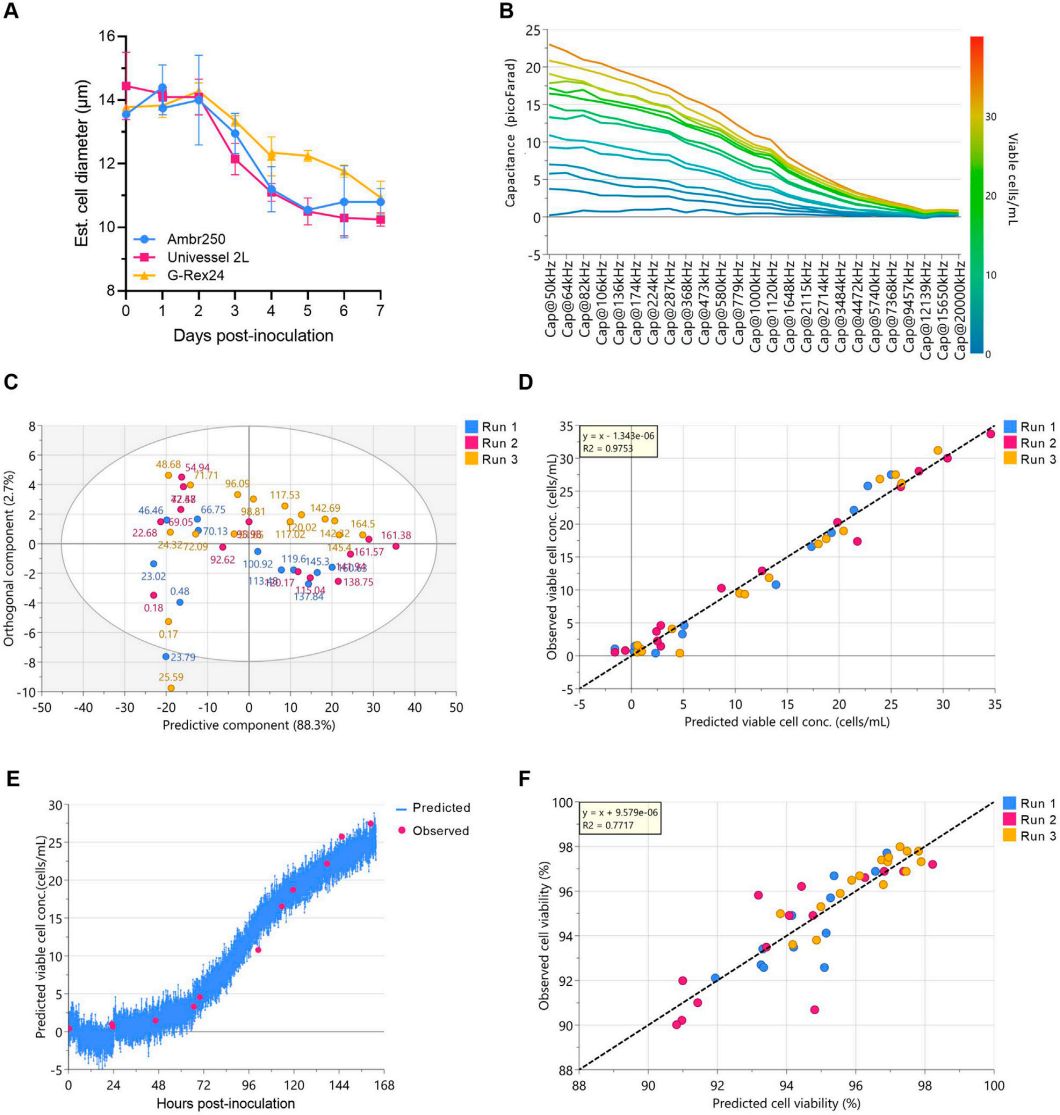
Multifrequency capacitance sensing enables accurate predictions of 2 L bioreactor CAR-T cell concentrations. **(A)** Daily estimated cell diameters (NucleoCounter® NC-3000™). Data presented as mean ± SD of n = 3 experimental replicates with one healthy donor with well-plate data presented as the mean of n = 3 technical replicate wells per experimental replicate (n = 9). **(B)** Multifrequency capacitance measurements in a representative 2 L Univessel® stirred-tank bioreactor (STR) culture, coloured by cell density determined from offline cell counts. Each line represents measurements taken at a specific time point corresponding to an offline cell count. **(C)** Principal component analysis score plot of multifrequency capacitance data from n = 3 2 L STR experimental replicates; each point labelled with offline measurement culture time (hours). The score plot ellipse represents the 95% confidence region based on Hotelling’s T^2^ value. **(D)** Linear regression of 2 L STR cell concentrations predicted via multivariate analysis of capacitance measurements versus offline cell counts. **(E)** Model prediction of cell concentrations (blue) for a representative 2 L STR experiment, overlaid with observed cell counts from offline sampling (pink). **(F)** Linear regression of 2 L STR cell viabilities predicted via multivariate analysis of capacitance measurements versus offline cell counts.

To enable and assess real-time monitoring of CAR-T cell concentrations, a capacitance probe was integrated into the 2 L STR process. Capacitance reflects the ability of an object to store electric charge. In viable cells, the intact phospholipid membrane acts as an insulator surrounding the conductive cytoplasm, allowing the cells to become polarised when exposed to an alternating electric field (Metze et al., 2020). This polarisation generates a measurable bio-capacitance signal that can be measured at one or multiple frequencies. Influenced by culture volume and the number, size, and morphology of viable cells, capacitance can be used to derive viable cell density. Single-frequency capacitance measurement relies on a fixed correlation with viable cell concentration and is therefore confounded by changes in cell size, such as those observed following T cell activation (Figure 4A). To address this, multifrequency scanning was used to improve the accuracy of viable cell density predictions.

The dielectric properties of CAR-T cell cultures were measured across 25 discrete frequencies (50–20,000 kHz) throughout the 7-day perfusion process in the 2 L STR. Figure 4B shows cell culture capacitance from a representative 2 L STR culture measured across all frequencies and colour-coded by viable cell density as determined from offline cell counts. Each of the 14 trendlines represents the multi-frequency capacitance measurement acquired at the time each offline sample was taken, with sampling performed twice daily over the 7-day cultures. Differences in capacitance were most pronounced when measured at lower frequencies, with higher cell densities (yellow/red lines) associated with elevated capacitance compared with lower-density samples (blue lines). At frequencies above 7,368 kHz, capacitance values from all timepoints converged toward similar levels, indicating reduced sensitivity of the measurement to cell density. At these higher frequencies only partial polarisation of the cell membrane occurs, resulting in a diminished capacitance signal (Bergin et al., 2022).

Principal component analysis (PCA) of capacitance data over 7 days from all three replicate 2 L runs showed time-based clustering of capacitance data (Figure 4C), consistent with observed viable cell density trends (Figure 2). To quantitatively relate capacitance measurements to cell densities, an orthogonal partial least squares (OPLS) regression model was developed using multi-frequency capacitance measurements as predictors and offline viable cell counts as responses. The resulting model demonstrated strong correlation to offline cell counts (R^2^ = 0.98) and high predictive performance (Q^2^ = 0.96) (Figure 4D).

Due to the low number of experimental runs (n = 3), model diagnostics were performed to assess the risk of overfitting and evaluate the reliability of the OPLS model. The cross-validated score plot showed overlapping predictive behaviour across the three bioreactor runs, indicating consistent model performance between experiments (Supplementary Figure S6a). The distance to model in X-space (DModX) was then calculated to identify potential X-outliers, with observations exceeding the critical limit (Dcrit = 0.05) considered as statistically significant outliers. All but two samples fell below this threshold, indicating that the capacitance spectra for most samples were well captured by the model and did not exhibit unusually large X-residuals (Supplementary Figure S6b). Hotelling’s T^2^ analysis similarly showed that all but one observation fell well within the 95% critical limits, demonstrating stable and consistent multivariate behaviour across runs without systematic outliers (Supplementary Figure S6c). Y-residuals followed a normal distribution, suggesting that prediction errors behaved like random noise rather than systematic model bias (Supplementary Figure S6d). Finally, permutation testing yielded lower R^2^ and Q^2^ values for the permuted models compared with the original model, confirming that the observed predictive structure was stronger than expected by chance and not an artefact of supervised overfitting (Supplementary Figure S6e). Together, these diagnostics demonstrated that the OPLS model exhibited consistent internal behaviour and reasonable predictive reliability within the constraints of a limited dataset (n = 3 runs).

To further assess model generalisability across bioreactor batches, the OPLS model was validated using a leave-one-batch-out approach, by training the OPLS model on two of the three bioreactor runs and predicting the third. This confirmed that the model retained strong predictive accuracy when applied to unseen bioreactor runs and that its performance was not dependent on any individual dataset (Figure 4E). Lastly, given that capacitance is partially influenced by cell viability, its potential for predicting this parameter was also assessed (Figure 4F). While regression showed a reasonable model fit (R^2^ = 0.78), the lower predictive power (Q^2^ = 0.62) indicated further model refinement would be required to enable reliable tracking of cell viabilities.

### 2.6 Comparable phenotype and cytotoxicity of CAR-T cells expanded in 250 mL vs. 2 L bioreactors

**FIGURE 5:**
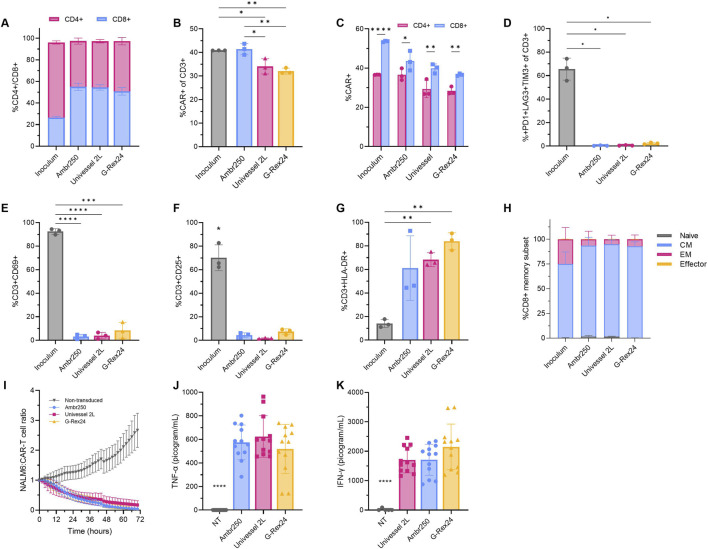
Comparison of CAR-T cell phenotype, cytotoxicity, and cytokine secretion between 250 mL and 2 L perfusion bioreactors and static well plates. Cellular phenotype was analysed via flow cytometry upon bioreactor inoculation and harvest. **(A)** CD4+/CD8+ ratio, **(B)** CAR expression on CD3+ and **(C)** CD4+ and CD8+ cells. **(D)** CD3+ exhaustion marker co-expression (PD-1, LAG-3, TIM-3), **(E)** CD3^+^ early activation (CD69), **(F)** mid-activation (CD25) and **(G)** late activation (HLA-DR) marker expression. **(H)** Proportion of CD8+ cells classified as naïve (CCR7+CD45RO−), central memory (CM) (CCR7+CD45RO+), effector memory (EM) (CCR7−CD45RO+), or effector (CCR7−CD45RO−). Data presented as mean ± SD of n = 3 experimental replicates using one donor with well plate data presented as the mean of n = 3 technical replicate wells per experimental replicate (n = 9). **(I)** Harvested CAR-T cell cytotoxicity was assessed *in vitro* via fluorescent microscopy by 1:1 co-culture with Nuclight Green NALM6 target cells. **(J)** Tumour necrosis factor-alpha (TNF-α) and **(K)** interferon-gamma (IFN-γ) cytokine secretion measured in cytotoxicity assay culture medium after 48 h. Data presented as mean ± SD of n = 4 technical replicates for each n = 3 experimental replicates (n = 12), using one healthy donor. Statistical analyses were performed using Welch’s ANOVA and Dunnett’s T3 multiple comparison test with *p < 0.05, **p < 0.01, and ***p < 0.001.

To compare final CAR-T product quality attributes from the different bioreactor processes, flow cytometry, cytotoxicity and cytokine secretion assays were performed with CAR-T cells harvested from the 250 mL and 2 L perfusion STRs and static well plates. Cell surface marker expression was evaluated at inoculation (Day 0) and harvest (Day 7), to analyse T cell subsets, CAR expression, exhaustion, activation and differentiation profiles (Figure 5).

In all processes, the CD4+/CD8+ ratio shifted from a CD4-dominant population (∼3:1) at inoculation to approximately 1:1 ratio by harvest (Figure 5A), driven by a consistent increase in the proportion of CD8^+^ cells over time. By harvest, final CD3+ CAR transduction efficiencies ranged from 32% to 41% across processes. In the 250 mL STR, the proportion of CAR+ cells remained comparable to that observed at inoculation (41.4% ± 2.4%), whereas proportions dropped significantly in the 2 L STR (33.6% ± 3.2%) and in static well plates (32.0% ± 1.3%) (Figure 5B). The reasons for these differential reductions in CAR expression were not fully understood, although they may relate to differences in the expansion kinetics of non-modified T cells between the processes. Further analysis of CAR expression in CD4+/CD8+ subsets revealed consistently superior transduction of CD8+ cells across all processes and time points (Figure 5C).

The proportion of exhausted cells, characterised by the co-expression of PD-1, LAG-3, and TIM-3 surface markers, decreased markedly by ∼60% from inoculation to harvest, resulting in comparably low final exhaustion levels of 3% in all conditions (Figure 5D). Activation marker expression profiles followed expected trends with expression of early (CD69) and mid-stage (CD25) markers declining sharply over time (Figures 5E,F), while late (HLA-DR) marker expression increased by harvest (Figure 5G). Interestingly, expression of early, mid, and late activation markers was higher in well plate cultures compared to STRs, correlating with larger average cell sizes (Figure 4A), and potentially indicating a more prolonged activation state in static culture. Differentiation of CD8+ T cells was assessed via expression of CCR7 and CD45RO to quantify the proportion of naïve (CCR7+CD45RO-), central memory (CCR7+CD45RO+), effector memory (CCR7-CD45RO+), and effector (CCR7-CD45RO-) subsets. By harvest, all bioreactor processes yielded comparable cellular differentiation profiles with more than 90% of harvested cells expressing either naïve or central memory markers and <10% expressing effector memory or effector markers (Figure 5H). Notably, the proportion of central memory CD8+ cells was found to increase by ∼24% by harvest in all processes.

To further assess functional *in vitro* cytotoxicity, CAR-T cells harvested from all platforms were purified and co-cultured for 3 days with Nuclight Green CD19^+^ NALM6 acute lymphoblastic leukaemia target cells. All conditions demonstrated equivalent, target-specific cytotoxicity relative to non-transduced T cell controls (Figure 5I), as confirmed by fluorescence microscopy (Supplementary Figure S7). Analysis of killing assay culture supernatants after 2 days further confirmed target-specific cytokine release, with comparable TNF-α and IFN-γ secretion by CAR-T cells (Figures 5J,K). Taken together, these results demonstrated CAR-T products generated in 250 mL and 2 L bioreactors, as well as static well plates, exhibited comparable anti-cancer cytotoxicity.

In summary, CAR-T cells expanded in both 250 mL and 2 L STRs exhibited comparable phenotypes and cytotoxic potency, supporting the consistency of final product quality across scales. Moreover, the similarity with static well plate cultures confirmed that perfusion-based expansion in STRs did not negatively impact CAR-T cell quality attributes.

### 2.7 Multivariate analysis confirms CAR-T product similarity between 250 mL and 2 L bioreactors

**FIGURE 6:**
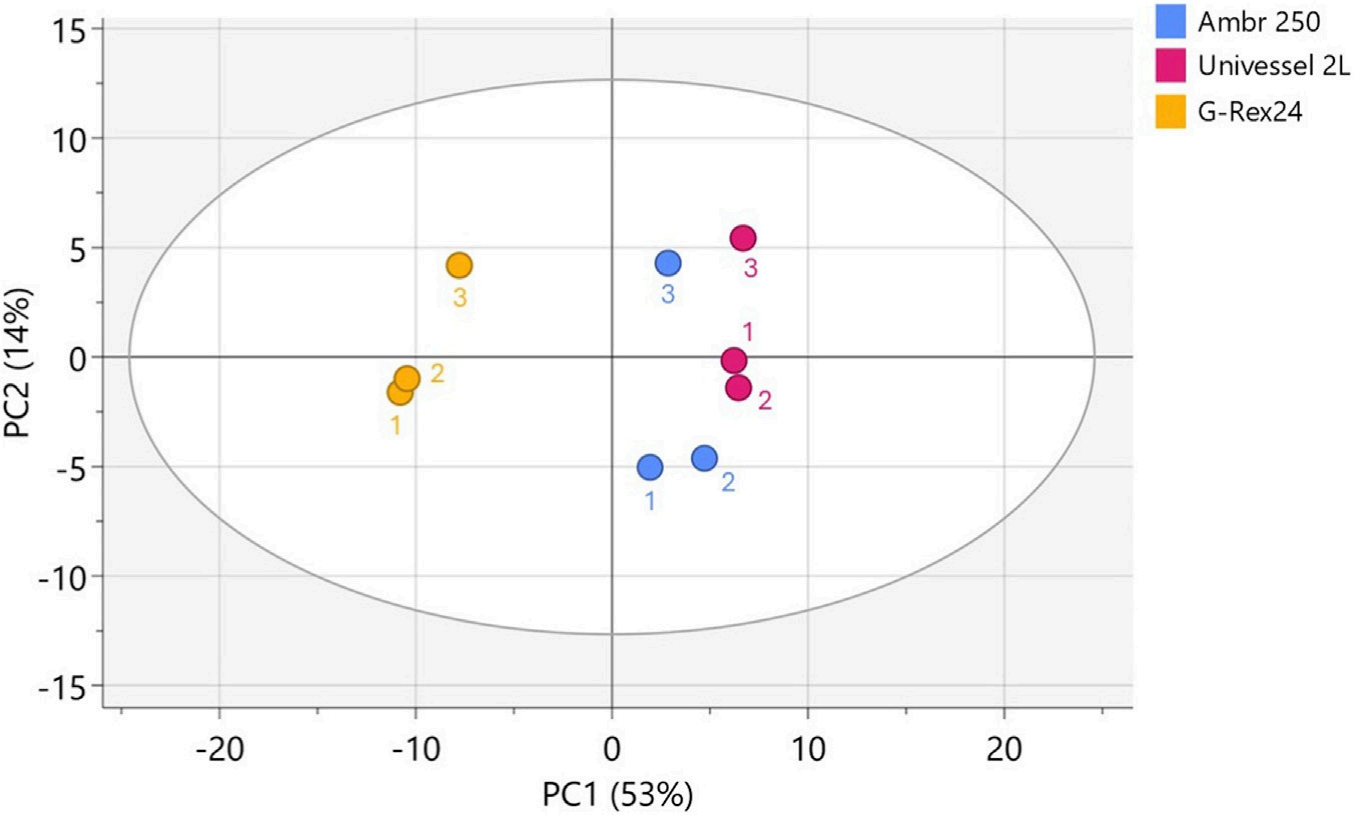
Comparable final CAR-T product quality attributes from 250 mL and 2 L perfusion bioreactors. Principal component analysis score plot of final CAR-T cells harvested Ambr® 250 and Univessel® 2 L perfusion stirred-tank bioreactors and G-Rex®24 well plates. Each point represents one experimental replicate (n = 3) and condenses all daily cell growth, viability, and metabolite data over the 7-day bioreactor expansion, along with all phenotypic, cytotoxicity, and cytokine release measurements taken at harvest (Day 7). Points are labelled according to their experimental replicate. The score plot ellipse represents the 95% confidence region based on Hotelling’s T^2^ value.

To holistically assess CAR-T product similarity between the 250 mL and 2 L STR processes and evaluate differences relative to static well plate cultures, all quality attribute data were integrated and analysed via PCA. Time-series data on cell growth, viability, glucose/lactate levels and metabolic rates, along with harvest quality attributes (phenotype, cytotoxicity, cytokine secretion), were summarised as single data points per run. In the resulting PCA score plot (Figure 6), clustering of points indicates statistical similarity across manufacturing conditions. A clear separation was observed between well plate and STR-derived CAR-T cells, driven largely by the inferior cell growth achieved in well plates (Figure 2), as supported by additional factor analysis (data not shown). In contrast, data from the 250 mL and 2 L bioreactors clustered closely, indicating higher statistical similarity of CAR-T product quality attributes overall. These findings supported earlier univariate results showing comparable cell growth and phenotype (Figures 2, 5), and substantiated that CAR-T cell quality was widely similar between both STR scales.

### 2.8 Automated 2 L bioreactor harvesting, product concentration and washing maintains CAR-T cell quality attributes

**FIGURE 7:**
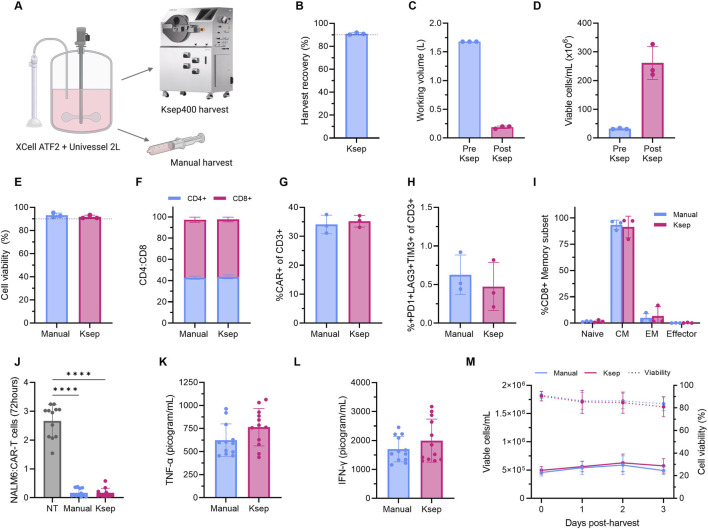
Automating 2 L bioreactor CAR-T product harvest, concentration and washing using the Ksep® 400 maintains product quality. **(A)** After 7-day expansion in the Univessel® SU 2 L bioreactor, CAR-T cells were harvested, concentrated and washed using the Ksep® 400 and compared to cells manually sampled via syringe. **(B)** Ksep® 400 harvest recovery efficiency, **(C)** pre- and post-harvest working volume, **(D)** viable cell concentration and **(E)** cell viability. Cellular phenotypes from manual and Ksep® 400 harvested cells were analysed by flow cytometry post-harvest. **(F)** CD4+/CD8+ ratio, **(G)** CAR expression on CD3+ cells, **(H)** CD3+ exhaustion marker co-expression (PD-1, LAG-3, TIM-3), and **(I)** Proportion of CD8+ cells classified as naïve (CCR7+CD45RO−), central memory (CM) (CCR7+CD45RO+), effector memory (EM) (CCR7−CD45RO+), or effector (CCR7−CD45RO−). Data presented as mean ± SD of n = 3 experimental replicates with one donor with well-plate data presented as the mean of n = 3 technical replicate wells per experimental replicate (n = 9). **(J)** Harvested CAR-T cell cytotoxicity was assessed *in vitro* via fluorescent microscopy by 1:1 co-culture with Nuclight Green NALM6 target cells. **(K)** Tumour necrosis factor-alpha (TNF-α) and **(L)** interferon-gamma (IFN-γ) cytokine secretion measured in cytotoxicity assay culture medium after 48 h. **(M)** Harvested cells were resuspended in fresh medium supplemented with IL-2 and expanded in static T-flasks for 3 days. Data presented as mean ± SD of n = 3 technical replicates for each n = 3 experimental replicates (n = 9), using one healthy donor. Statistical analyses were performed using t-tests and Welch’s ANOVA and Dunnett’s T3 multiple comparison test with *p < 0.05, **p < 0.01, and ***p < 0.001.

The impact of automating bioreactor harvest, CAR-T cell concentration, and washing using counterflow centrifugation (Ksep® 400) was evaluated at the end of the 2 L STR process. Post-harvest CAR-T cell viability, phenotype, cytotoxicity, and proliferative capacity were compared to control cells manually sampled from the bioreactor using a syringe (Figure 7A).

The Ksep® 400 system uses counterflow centrifugal elutriation, in which the bioreactor cell culture is pumped against centrifugal forces to concentrate harvested cells into a fluidised bed while allowing elutriation of the culture medium. The fluidised bed of cells was subsequently washed with phosphate-buffered saline (PBS), resulting in a concentrated final product. The automated harvest, concentration and washing of the full 2 L bioreactor cell cultures was completed within 30–35 min, achieving a recovery efficiency of 91.0% ± 1.0% with high reproducibility across runs (Figure 7B). Sampling of waste fractions confirmed small levels of cellular losses which accounted for the incomplete recovery rates. The total 1.68 L bioreactor culture volume was concentrated to 186.7 ± 23 mL, representing a 9-fold volume reduction, with final product concentrations reaching 261 ± 46.7 × 10^6^ cells/mL (Figures 7C,D). Post-harvest cell viabilities remained statistically unchanged remaining at 91% ± 1.5%, indicating that centrifugation, peristaltic pumping and concentration steps were well tolerated (Figure 7E). Similarly, levels of CAR transduction levels, cellular exhaustion and maturation, cytotoxicity, and cytokine secretion also remained statistically comparable between manually and Ksep® 400-processed cells confirming that automated downstream processing had no adverse impacts on CAR-T product quality attributes (Figures 7F–L).

To further assess potential effects of automated harvesting on cellular proliferative capacity, harvested cells were cultured for an additional 3 days in T-flasks with fresh IL-2–supplemented medium. Post-harvest proliferation was comparable between manually and Ksep® 400–harvested cells, with both conditions consistently showing minimal expansion and a gradual decline in viability to 81%–84%, indicating that automated processing and concentration did not adversely impact cell proliferation (Figure 7M). Interestingly, this post-harvest growth arrest contrasted significantly with the continued proliferation observed from Days 7–10 in extended 250 mL perfusion cultures (Supplementary Figure S3b). This suggested that the presence of endogenous factors other than IL-2 remaining in the bioreactor environment, but potentially lost by resuspension into fresh medium in T-flasks, may have contributed to sustaining CAR-T cell proliferation in the extended 250 mL bioreactor cultures.

Together, these results demonstrated that automated CAR-T cell product harvesting, concentration and washing via the Ksep® 400 was efficient, reproducible and did not impact final CAR-T product quality attributes.

## 3 Discussion

### 3.1 Stirred-tank bioreactors for large-scale CAR-T production

The emergence of allogeneic, universal CAR-T therapies presents a promising opportunity to overcome key limitations of autologous production, including variable starting material, complex logistics, long lead times, and high per-dose costs (Piscopo et al., 2018; Dias et al., 2024). By enabling off-the-shelf availability and large-batch manufacturing, allogeneic therapies have the potential to accelerate and expand patient access alongside significantly reducing costs per dose. In the recent allogeneic CAR-T ALPHA and ALPHA2 Phase I trials, for example, the reported median time from patient enrolment to treatment was just 2 days (Locke et al., 2025), marking a substantial improvement over the typical 4–6 weeks required for autologous CAR-T products, highlighting the potential of allogeneic therapies to support more timely intervention for patients often facing rapid disease progression (Depil et al., 2020). To fully realise this potential, however, intensified and scalable manufacturing processes capable of producing high yields of high-quality CAR-T cells at economies of scale will be essential.

To date, clinical production of allogeneic CAR-T cells has primarily relied on rocking bioreactors due to their immediate availability in single-use formats, low-shear and legacy use in autologous CAR-T manufacturing (Qasim et al., 2017; Sugita et al., 2022; Georgiadis et al., 2025). Although rocking bioreactors have been used for CAR-T cell cultures up to the 10 L scale (Sugita et al., 2022), their scalability is limited. At larger volumes, performance may be hindered by poor mixing, restricted oxygen transfer due to surface area-to-volume constraints, and mechanical limitations inherent to the rocking motion (Bartczak et al., 2022). In contrast, STRs offer superior mass transfer via impellor-based agitation and proven scalability beyond 20,000 L (Nienow, 2014; Manahan et al., 2019). They can be operated in batch, fed-batch, and perfusion modes, integrate well with PATs, and have been extensively characterised with a strong record in the commercial production of monoclonal antibodies (Manahan et al., 2019), vaccines (Fang et al., 2022), and viral vectors (Andaluz et al., 2025). These attributes therefore position STRs as robust and scalable platforms that are well suited to achieve the projected 200–2,000 L allogeneic batch capacities needed to meet future allogeneic therapy demand (Pigeau et al., 2018).

Concerns about the shear sensitivity of T cells and immune cells more generally have historically limited the adoption of STRs. However, here we report a CAR-T cell expansion process intensified via perfusion that successfully achieved 30–47 × 10^6^ cells/mL within 7–10 days at the 250 mL scale. When performed at the 2 L scale, this process consistently yielded 113 ± 7 functional CAR-T doses per batch within 7 days of expansion. In both bioreactor scales, cell viabilities were maintained comparably high above 90% in serum-free medium, despite exposure to shear-related stresses including peristaltic pumping, impeller mixing, oxygen sparging, and ATF perfusion. These results add to the increasing demonstrations of T and CAR-T cell expansions in STRs that confirm the resilience of these cells under well-optimised conditions and the suitability of STR platforms to scale-up CAR-T manufacturing (Ou et al., 2019; Costariol et al., 2020; Gatla et al., 2022; Lamas et al., 2022; Selvarajan et al., 2024; Costa et al., 2025; Hood et al., 2025; Silva Couto et al., 2025; Springuel et al., 2025).

While previous studies have demonstrated T cell expansion in STRs up to 1 L (Ou et al., 2019; Gatla et al., 2022), this study is the first to characterise CAR-T cell-specific quality attributes at the multi-litre scale. We report stable CAR transgene expression throughout the 2 L STR expansion process, with final product retaining comparable *in vitro* cytotoxicity as static well plate controls. Over 90% of harvested CD8^+^ cells exhibited naïve or central memory phenotypes, with minimal exhaustion marker expression, characteristics that been associated with improved in vivo efficacy and persistence (Fraietta et al., 2018; Shah and Fry, 2019; Deng et al., 2020). These results support the feasibility of multi-litre, STR-based perfusion process to produce large numbers of high-quality CAR-T cell doses. While conducting this study in n = 3 technical replicates using a single donor enabled the assessment of batch-to-batch process consistency and reproducibility, additional studies incorporating multiple donors would be required to confirm process robustness across broader biological variability. Moreover, since this study only utilised autologous anti-CD19 CAR-T cells, future work should also extend the presented workflow to allogeneic CAR-T cells to assess relevant quality attributes such TCR/HLA silencing and final CAR-T product alloreactivity. In parallel, further process development efforts aimed at integrating additional upstream process steps such as cell thaw, activation, and transduction directly into STRs could enable a more streamlined all-in-one manufacturing workflow. This would facilitate process translation into GMP environments by eliminating the manual and open flask and centrifugation steps used in this study prior to bioreactor inoculation. Additionally, the CAR-T dose yields reported in this study only reflect the total bulk product available at the end of the bioreactor expansion phase. Potential product losses occurring over harvest, concentration, and formulation steps and usage for quality control were not accounted for. Future analyses that incorporate product losses across a complete downstream processing workflow would provide more representative whole-process CAR-T dose yield estimates.

Future large-scale CAR-T production will also require the integration of advanced PATs to accurately monitor critical process parameters such as CAR-T cell concentrations within the bioreactor in real time (Aifuwa, 2022). Conventional methods have relied on manual sampling and offline cell counting which introduces contaminations risks and valuable product loss over time. While capacitance-based biomass sensing is well established in antibody production workflows, its application to CAR-T processes has so far been limited. Here, we demonstrate for the first time the suitability of capacitance sensing for real-time, *in situ* monitoring of CAR-T cell concentrations, showing a strong correlation with offline viable cell counts at the 2 L scale (R^2^ = 0.98). The use of multi-frequency spectroscopic scanning over single-frequency approaches is likely to be essential to account for dynamic changes in CAR-T cell diameter that occur following cell activation (Waugh et al., 2023). Single-frequency capacitance measurements primarily reflect the polarisation of viable cell volume, making them sensitive to shifts in cell size over time (Metze et al., 2020). In contrast, scanning across multiple frequencies, combined with multivariate data analysis, enables more robust correlation with viable CAR-T cell concentrations and maintains accuracy throughout the expansion process despite changes in T cell morphology post-activation.

Beyond eliminating the need for manual sampling and improving process monitoring, the integration of capacitance sensing provides an opportunity to enhance CAR-T process control by automating adjustments of critical process parameters such as perfusion rates based on cell growth in real-time (Bergin et al., 2022). Automated perfusion rate control based on capacitance measurements has successfully been implemented to improve viral vector and antibody yields, while also reducing overall medium consumption by 15% (Nikolay et al., 2018; Müller et al., 2022; Olin et al., 2024; Sun et al., 2025). Applying similar perfusion control in large-scale allogeneic CAR-T manufacturing to automatically match perfusion rates to the rise and fall of CAR-T cell growth rates post-activation could help minimise medium consumption and overall costs.

### 3.2 Stirred-tank bioreactors to facilitate quality by design CAR-T process development

A key limitation of conventional CAR-T manufacturing platforms is the lack of validated scale-down models (SDMs) that can accurately replicate manufacturing-scale conditions, enabling high-throughput screening of critical process parameters and their effects on product quality at small-scale and lower costs (Sandner et al., 2018). In this study, we validated the Ambr® 250 HT as a representative SDM capable of predicting both process performance and CAR-T product outcomes observed in the 2 L Univessel®. Due to similar geometries and fluid dynamics based on matched P/V, highly comparable process performance and CAR-T quality attributes were achieved between the 250 mL and 2 L scales as confirmed by univariate and multivariate analyses of cell yields, phenotype, cytotoxicity and cytokine secretion. This approach reflects established practices in commercial biopharmaceutical manufacturing, where representative SDMs are routinely used to support commercial process development and optimisation (Kochanowski and Malphettes, 2013).

Prior to this study, we leveraged the Ambr® 250 HT system to extensively characterise and develop an optimised perfusion process which enabled the seamless scale-up to 2 L demonstrated here (Hood et al., 2025; Springuel et al., 2025). Similarly, the smaller Ambr® 15 system (15 mL) was recently used to scale-up an adeno-associated virus production process to 2,000 L under 2 months (Andaluz et al., 2025). SDM-based strategies therefore offer allogeneic CAR-T therapy developers a powerful platform to efficiently evaluate parameters including cell source, media, cytokines, environmental controls, and perfusion regimes at small scale and high-throughput. This approach can enable the optimisation of process productivity, product quality and costs of goods while accelerating and de-risking scale-up to clinical or commercial scales. Critically, SDM-driven process characterisation aligns with the FDA’s longstanding QbD principles for pharmaceutical production outlined in the ICH Q8 (R2) guidance and supports the level of process understanding recently emphasised in FDA guidance on the development of CAR-T cell products. Moreover, when integrated with robotics, machine learning, and digital modelling, small-scale, parallelised bioreactor systems such as the Ambr® 250 HT are well suited to accelerate emerging trends in autonomous, self-optimising process development, as recently demonstrated in perfusion-based antibody production (Morschett et al., 2021; Müller et al., 2024).

### 3.3 Considerations for scalable CAR-T cell downstream processing

The shift toward large-batch CAR-T production will necessitate downstream solutions that are equally scalable to efficiently process multi-litre product batches. In this study, we demonstrate that the Ksep® 400 counterflow centrifugation system successfully automated the harvest, washing, and concentration of CAR-T cells from the 2 L bioreactor, recovering over 90% of total viable cells while achieving a 9-fold volume reduction, making subsequent downstream processing more manageable. This recovery efficiency exceeds previously reported values at the 2 L scale with T cells (69%) (Gatla et al., 2022), highlighting how the optimisation of parameters such as flow rates, centrifugal forces, and processing times can significantly enhance harvest efficiencies. Importantly, the automation of these downstream operations was not found to have any detrimental impacts on CAR-T cell viability, phenotype, or cytotoxicity demonstrating its suitability as a scalable and automated solution for large-scale CAR-T manufacturing. While this study utilised PBS to wash and concentrate the harvested CAR-T product, incorporating final cryopreservation formulations directly into this workflow instead, could further streamline final product preparation by eliminating the need for a separate formulation unit operation. Future downstream development efforts should now focus on scalable formulation and filling solutions to enable efficient and scalable packaging of large numbers of CAR-T doses into final product bags suitable for freezing, storage, and transport to treatment centres.

## 4 Conclusion

This work demonstrates the scalable expansion of CAR-T cells to high densities in serum-free medium using perfusion in multi-litre, single-use STRs, enabling the generation of over 110 functional doses per batch. Counterflow centrifugation was demonstrated as a scalable downstream operation to streamline and automate CAR-T product harvest, concentration, and washing into a single unit operation at the multi-litre scale, with no adverse impact on product quality. Capacitance sensing was validated for the real-time monitoring of CAR-T cell concentrations in the 2 L STR, providing future opportunities to improve perfusion process control, and the Ambr® 250 was characterised as a representative SDM which can significantly support allogeneic CAR-T process development and de-risk scale-up. Together, the presented workflow offers a robust and scalable manufacturing platform to meet future demand for allogeneic therapies and helps advance CAR-T manufacturing towards the well-established standards of large-scale commercial biologics production.

## 5 Methods

### 5.1 Lentiviral vector production and titration

A second-generation lentiviral vector encoding an anti-CD19 CAR was produced via plasmid-transfection of adherent HEK293 cells (ATCC) cultured in DMEM medium (Thermo Fisher Scientific) supplemented with 10% fetal bovine serum (FBS) (Thermo Fisher Scientific) in 10-layered flasks (Corning). Six hours post-transfection, a fresh medium exchange was performed and cells were incubated for 48 h. The virus-containing supernatant was harvested and filtered using 0.45 µm filters (Thermo Fisher Scientific) and aliquoted and stored at −80 °C until experimentation.

The produced virus was titrated via serial dilution and transduction of 1.5 × 10^6^ Jurkat cells (ATCC) in Retronectin-coated (Takara Bio) well plates and spinoculation at 1000 g for 40 min at 32 °C. The level of CAR expression was quantified via flow cytometry 48 h post-transduction.

### 5.2 T cell isolation, activation, transduction and pre-expansion

Primary T cells were isolated from a single fresh, commercially available leukopak (BioIVT) by negative selection using Pan T cell magnetic separation kits (Miltenyi Biotec), and cryopreserved in CryoStor® CS10 (Sigma-Aldrich) at 50 × 10^6^ cells/mL in liquid nitrogen. For experiments, cells were thawed in xeno- and serum-free 4Cell® Nutri-T GMP medium (Sartorius) and rested overnight at 2 × 10^6^ cells/mL. The next day, cells were diluted to 1 × 10^6^ cells/mL, activated with anti-CD3/CD28 Dynabeads (Thermo Fisher Scientific) in a 1:1 bead-to-cell ratio and 30 IU/mL IL-2 (Miltenyi Biotec). One day post-activation, cells were plated in Retronectin-coated (Takara Bio) 6-well plates supplemented with IL-2, and transduced with lentiviral vector (MOI = 3) via spinoculation at 1000 g for 40 min at 32 °C. Following overnight incubation, cells were pooled and seeded in fresh medium at 0.5 × 10^6^ cells/mL in static T-flasks (Sarstedt) with IL-2 for 48 h of pre-expansion prior to bioreactor inoculation.

### 5.3 250 mL stirred-tank bioreactor preparation and operation

One day prior to inoculation, the Ambr® 250 High Throughput Perfusion system (Sartorius) was sterilised, and single-use perfusion vessels were installed. Bottles of fresh medium and 1 M sodium bicarbonate, fluid lines were primed, and vessels filled to hydrate pH and DO sensors overnight. On the day of inoculation, offline pH calibration was performed (Mettler Toledo), and 50 × 10^6^ total pre-expanded CAR-T cells were seeded into the bioreactor in 100 mL of Nutri-T medium. Agitation was maintained at 200 RPM, pH controlled at 7.35, and DO allowed to drop naturally before being maintained at 50% via headspace gassing of O_2_, N_2_ and CO_2_. One day post-inoculation, working volume was increased by by 110% to 210 mL, and ATF perfusion was initiated on day two at one vessel volume exchanges per day (VVD). Fresh medium perfusions were supplemented with 30 IU/mL IL-2, and medium bottles and permeate bags were exchanged as needed.

### 5.4 2 L stirred-tank bioreactor preparation and operation

One day before inoculation, a sterilised capacitance probe (Aber Instruments) and perfusion dip tube (Repligen) were installed in a Univessel® 2 L Single-Use bioreactor (Sartorius), mounted on a benchtop stand, and connected to the Biostat® B-DCU controller (Sartorius). The dip tube was linked to an XCell® ATF2 perfusion device via sterile connectors and the XCell® lab controller (Repligen). Bottles for permeate, fresh medium, and 1 M sodium bicarbonate were connected using a Biowelder® TC (Sartorius). The bioreactor was filled with 0.5 L medium overnight to hydrate pH/DO sensors and prime the ATF2 filter.

On inoculation day, pH calibration was performed, and 400 × 10^6^ pre-expanded CAR-T cells were seeded into 0.8 L Nutri-T medium. Agitation was set to 175 rpm to match the P/V in the Ambr® 250 bioreactor process which was operated at 200 rpm. pH was maintained at 7.35 and DO allowed to drop naturally before being maintained at 50% via headspace gassing of O_2_, N_2_ and CO_2_ at volumetric flow rates equivalent to the Ambr® 250 process. From Day 3 onwards, sparging of pure oxygen was added to the DO control cascade as required. One day post-inoculation, volume was increased by 110% to 1.68 L and perfusion was initiated on day two at 1.0 VVD, with fixed ATF2 flow rates of 650 mL/min to match Ambr® 250 perfusion shear. Medium exchange was controlled by standalone peristaltic pumps (Watson Marlow) with bottle weights monitored to ensure accurate flow.

### 5.5 Hydrodynamic scale-up characterisations

The agitation rate in the 2 L STR was scaled from our previously established 250 mL STR process by maintaining an equivalent volumetric power input (*P/V*) (W/m^3^), calculated using Equation 1, where $N_{p}$ is the previously reported power number (dimensionless) for the 250 mL (Rotondi et al., 2021) and 2 L STR (Zanghi et al., 2017), ρ is the density of the culture medium (assumed to be that of water at 37 °C = 993 kg/m^3^), N is the impeller agitation rate (s^-1^), D is the impeller diameter and V is the medium volume (m^3^).

$$\frac{P}{V}=\frac{N_{p}\times \rho \times N^{3}\times D^{5}}{V}$$ (1)

The average shear rate ( γ ) (s^-1^) produced by the STR impeller blades was calculated using Equation 2, where μ is the culture medium dynamic viscosity (assumed to be that of water at 37 °C = 6.91 × 10^−4^ Pa/s).

$$\gamma ={\left(\frac{P}{\mu \times V}\right)}^{\frac{1}{2}}$$ (2)

The Reynolds number ( Re ) (dimensionless) was calculated using Equation 3, tip speed ( $v_{tip}$ ) (m/s) using Equation 4 and Kolmogorov length scale ( η ) (m) using Equation 5 in which ε is the mean energy dissipation rate (W/kg).

$$Re=\frac{\rho \times N\times D^{2}}{\mu }$$ (3)

$$v_{tip}=\pi \times N\times D$$ (4)

$$\eta ={\left(\frac{\mu^{3}}{\rho^{3}\times \varepsilon }\right)}^{\frac{1}{4}}$$ (5)

A summary of bioreactor and impeller geometries, alongside calculated hydrodynamic characteristics for the 250 mL and 2 L STRs used in this study are provided in Table 2.

**TABLE 2:** Overview of stirred-tank bioreactor hydrodynamic parameters.

Parameter	Ambr® 250	Univessel® 2 L
Impeller type	1x elephant ear (45° pitch)	2x 3-blade segment (30° pitch)
Impeller diameter (m)	0.03	0.054
Impeller power number (−)	2.07	1.3
Medium volume (L)	0.21	1.68
Volumetric power input (W/m^3^)	8.81	8.75
Agitation rate (rpm)	200	175
Average shear rate (s^-1^)	113	113
Reynolds number (−)	4,311	12,222
Impeller tip speed (m/s)	0.31	0.49
Energy dissipation rate (W/kg)	0.00887	0.00882
Kolmogorov length scale (μm)	78.1	78.2

The ATF2 flow rate at the 2 L scale was selected to maintain an equivalent average shear rate ( γ ) (s^-1^) to that of the ATF perfusion filter of the Ambr® 250 bioreactor. Shear rate was calculated using Equation 6 where q is flow rate (m^3^/s) within the fiber lumen and r is the lumen radius (m). In the Ambr® 250 system, a perfusion cross-flow rate of 70 mL/min corresponded to an average shear rate of approximately 1,500 s^-1^, as provided by the manufacturer (Sartorius). Given that the ATF2 module contains 75 hollow fibers with 1 mm inner diameter, a total ATF2 flow rate of 650 mL/min was selected for the 2 L STR, which distributed across the 75 fibers yields a comparable per-fiber shear rate of ∼1,500 s^-1^.

$$\gamma =\frac{4\times q}{\pi {\times r}^{3}}$$ (6)

### 5.6 Capacitance multifrequency scanning and data analysis

A 12 mm Futura capacitance probe (Aber Instruments) installed in the Univessel® 2 L Single-Use bioreactor measured culture capacitance at 20 frequencies (50–20,000 kHz) using the Futura Scada software (Aber Instruments). Reference offline cell counts were performed twice daily throughout the 7-day 2 L bioreactor cultures. Collected capacitance data were imported into SIMCA v18 (Sartorius) and analysed using MVDA, with mean-centred capacitance values serving as predictive X-block variables and offline cell counts scaled to unit variance as Y-block response variables for viable cell concentration and viability predictive models. Capacitance values measured at frequencies >7,368 kHz were excluded from subsequent predictive OPLS modelling due to convergence of capacitance signals irrespective of viable cell concentration. A linear regression was applied and outliers identified using score plots in combination with distance to model in X-space (DModX) using a critical limit (Dcrit = 0.05), and Hotelling’s T^2^ with 95% and 99% critical limits. Normality of Y residuals was checked and model accuracy confirmed via permutation test. Lastly, OPLS model was validated using a leave-one-batch-out approach, by training the OPLS model on two of the three bioreactor runs and predicting the third.

### 5.7 Static well plate cultures

On the day of inoculation, 1 × 10^6^ total pre-expanded cells were seeded in 8 mL of Nutri-T medium supplemented with 30 IU/mL IL-2 per well of a G-Rex® 24-well plate (Wilson Wolf). IL-2 was replenished every 2 days, and a 75% medium exchange was performed on Day 4 without disturbing settled cells. Daily sacrificial sampling was performed for cell counts and metabolite analyses.

### 5.8 Automated bioreactor harvest, washing and concentration

At the end of the 7-day culture the Univessel® 2 L bioreactor was harvested using the Ksep® 400 via counterflow centrifugation. Single-use centrifugation chamber and tubing sets were installed in the Ksep® 400 and sterile-welded to an empty harvest bottle and a bottle containing phosphate-buffered saline (PBS) (Thermo Fisher Scientific). After priming with PBS, the Ksep® 400 consumable was connected to the 2 L bioreactor via sterile welding. Bioreactor harvest was initiated via counterflow centrifugation at 1000 g, concentrating cells into a fluidised bed at 60 mL/min. Once the entire bioreactor working volume was harvested, the fluidised bed of cells was washed and concentrated into a targeted volume of 200 mL of PBS at 200 mL/min. Final harvest volume and cell concentration were measured to calculate harvest recovery efficiencies.

### 5.9 Cell count and metabolite analysis

All bioreactor experiments were sampled daily for cell count, viability and estimated cell diameter analysis using the NucleoCounter® NC-3000™ cytometer (ChemoMetec). Following cell counting, cell-free supernatant was obtained via centrifugation and stored at −80 °C until later analysis of glucose and lactate concentrations using the CuBiAn HT270 analyser (4BioCell).

### 5.10 Flow cytometry

Cell surface marker expression was characterised on the day of bioreactor inoculation and harvest via flow cytometry. Analysis of T-cell and differentiation subset was performed with the LSRFortessa™ X-20 flow cytometer (BD Biosciences) using LIVE/DEAD™ stain (Thermo Fisher Scientific), BUV395 anti-CD3, BUV805 anti-CD4, APC-Cy7 anti-CD8a, BV421 anti-CCR7 and PE-Cy7 anti-CD45RO (BD Biosciences). Data was analysed using FlowJo™ v10 (FlowJo).

Analysis of T-cell activation and exhaustion was performed with the iQue®3 High Throughput Screening Cytometer (Sartorius) using the iQue® Human T Cell Activation (CD25, CD69, HLA-DR) and Exhaustion Kits (TIM-3, PD-1, LAG-3) according to the supplier’s instructions (Sartorius).

### 5.11 I*n vitro* cytotoxicity assay

Harvested CAR-T cells were resuspended in fresh medium at 2 × 10^6^ cells/mL and rested overnight. The following day, CAR + cells were purified via magnetic separation using CD34 microbead kits (Miltenyi) and 15,000 CAR-T cells were co-cultured 1:1 with Incucyte Nuclight Green-positive NALM6 CD19^+^ acute lymphoblastic leukaemia cells (ATCC) in 96-well plates and analysed using the Incucyte® S3 Live-Cell Analysis System (Sartorius) over 3 days. Four replicate 20X microscopy images were taken per well every 2 hours and NALM6 cell growth analysed using the Incucyte® S3 software. Supernatant samples were taken after 48 h and stored at −80 °C until analysis for cytokine secretion.

### 5.12 Cytokine secretion analysis

Secretion of tumour necrosis factor (TNF) alpha and interferon (IFN) gamma cytokines from CAR-T cells was analysed in medium samples from the cytotoxicity using the Human T Cell Cytokine Profiling kit (Sartorius) and iQue®3 High Throughput Screening Cytometer (Sartorius) according to the supplier’s instructions.

### 5.13 Statistical analyses

Principal component analysis (PCA) and orthogonal partial least squares (OPLS) were performed in SIMCA v18 (Sartorius). Data figures were generated and analysed in GraphPad Prism 9 (GraphPad), using t-tests and Welch’s ANOVA and Dunnett’s T3 multiple comparison test as required. Overview figures were created using BioRender (BioRender).

## Data Availability

The raw data supporting the conclusions of this article will be made available by the authors, without undue reservation.
