# Supplementary material for: Scalable CAR-T production in a 2-litre perfusion stirred-tank bioreactor with automated harvesting and scale-down model characterisation
Source: Front Bioeng Biotechnol. 2026 Jan 12;13:1694134. doi: 10.3389/fbioe.2025.1694134 (PMC12833271; doi:10.3389/fbioe.2025.1694134)
Supplement: Supplementary file 1 [file Supplementaryfile1.docx]

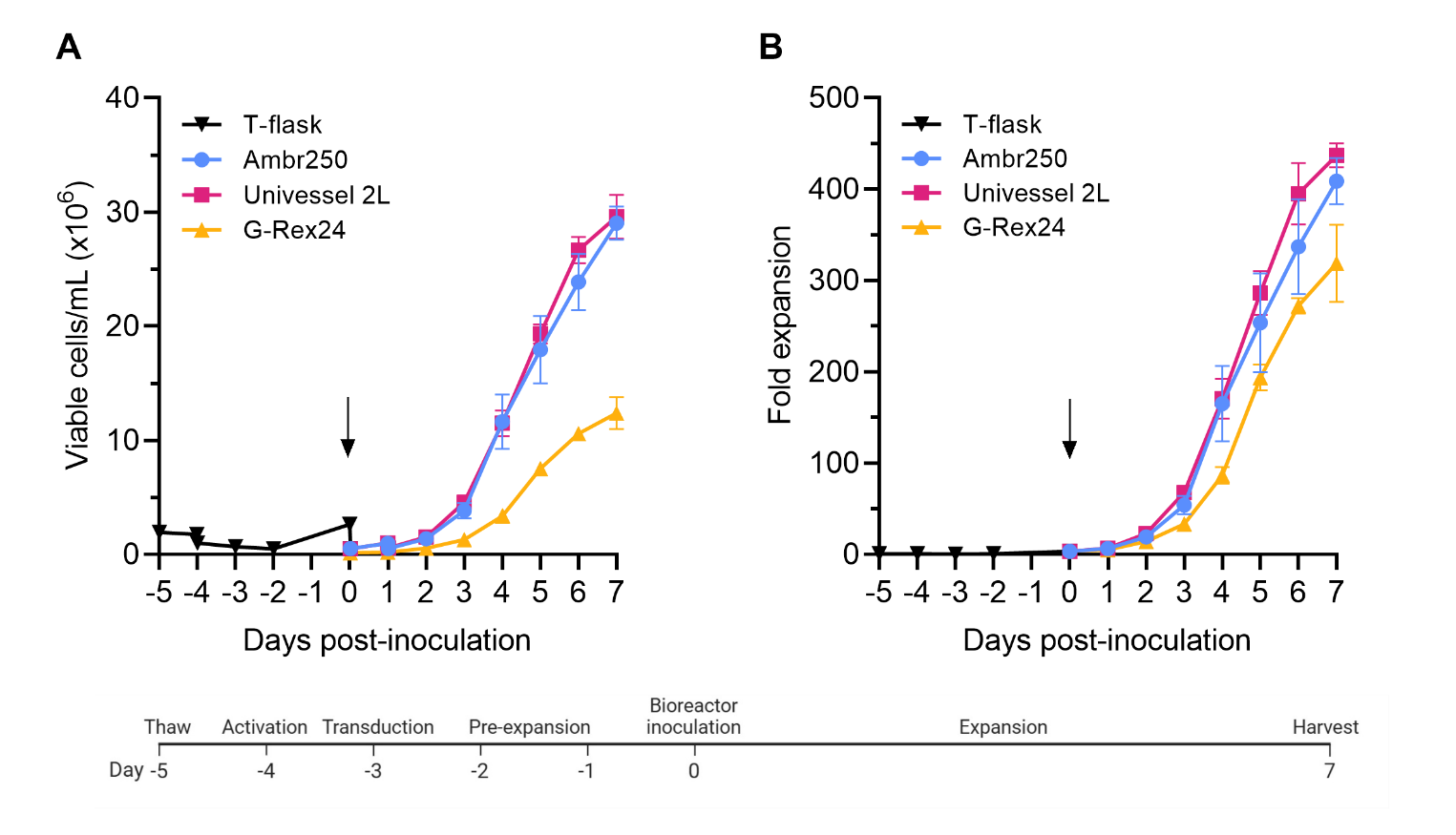
**Figure S1: Complete overview of cell expansion from thaw to bioreactor harvest.** Following lentiviral transduction on Day -3, anti-CD19 CAR-T cells were pre-expanded in static T flasks from Days -2-0 and inoculated in the Ambr^®^ 250 and Univessel^®^ 2 L stirred-tank bioreactors and G-Rex^®^24 well plates in parallel on Day 0 and expanded for 7-days**. a)** Daily viable cell densities and **b)** daily fold expansions. Data presented as mean ± SD of n=3 experimental replicates using one healthy donor with well plate data presented as the mean of n=3 technical replicate wells per experimental replicate (n=9). Arrow marks bioreactor inoculation.


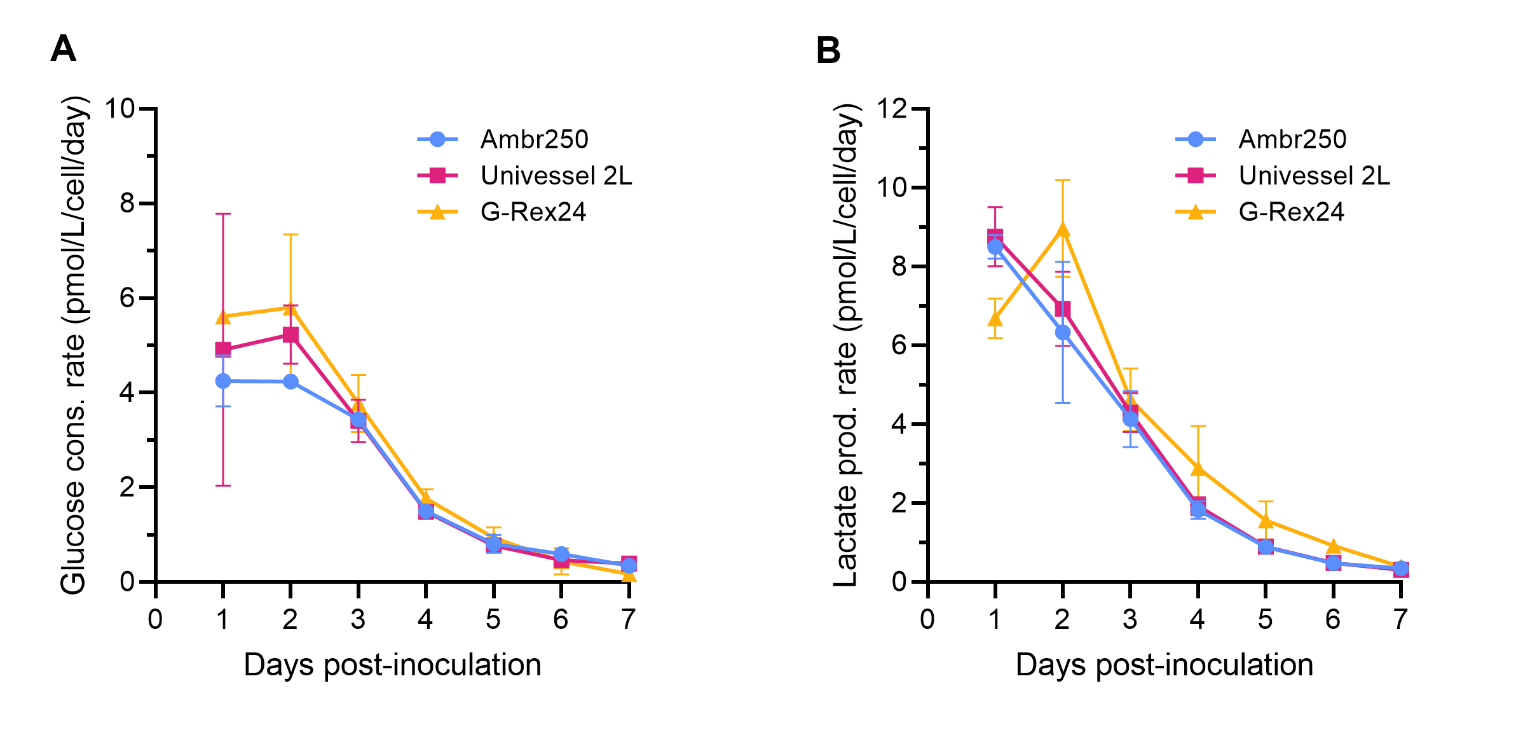

**Figure S2: CAR-T cell metabolic rates throughout bioreactor expansions.**Anti-CD19 CAR-T cells were inoculated in the Ambr^®^ 250 and Univessel^®^ 2 L stirred-tank bioreactors and G-Rex^®^24 well plates in parallel and expanded for seven days. **a)** Daily glucose consumption rates and **b)** daily lactate production rates. Data presented as mean ± SD of n=3 experimental replicates using one healthy donor with well plate data presented as the mean of n=3 technical replicate wells per experimental replicate (n=9).


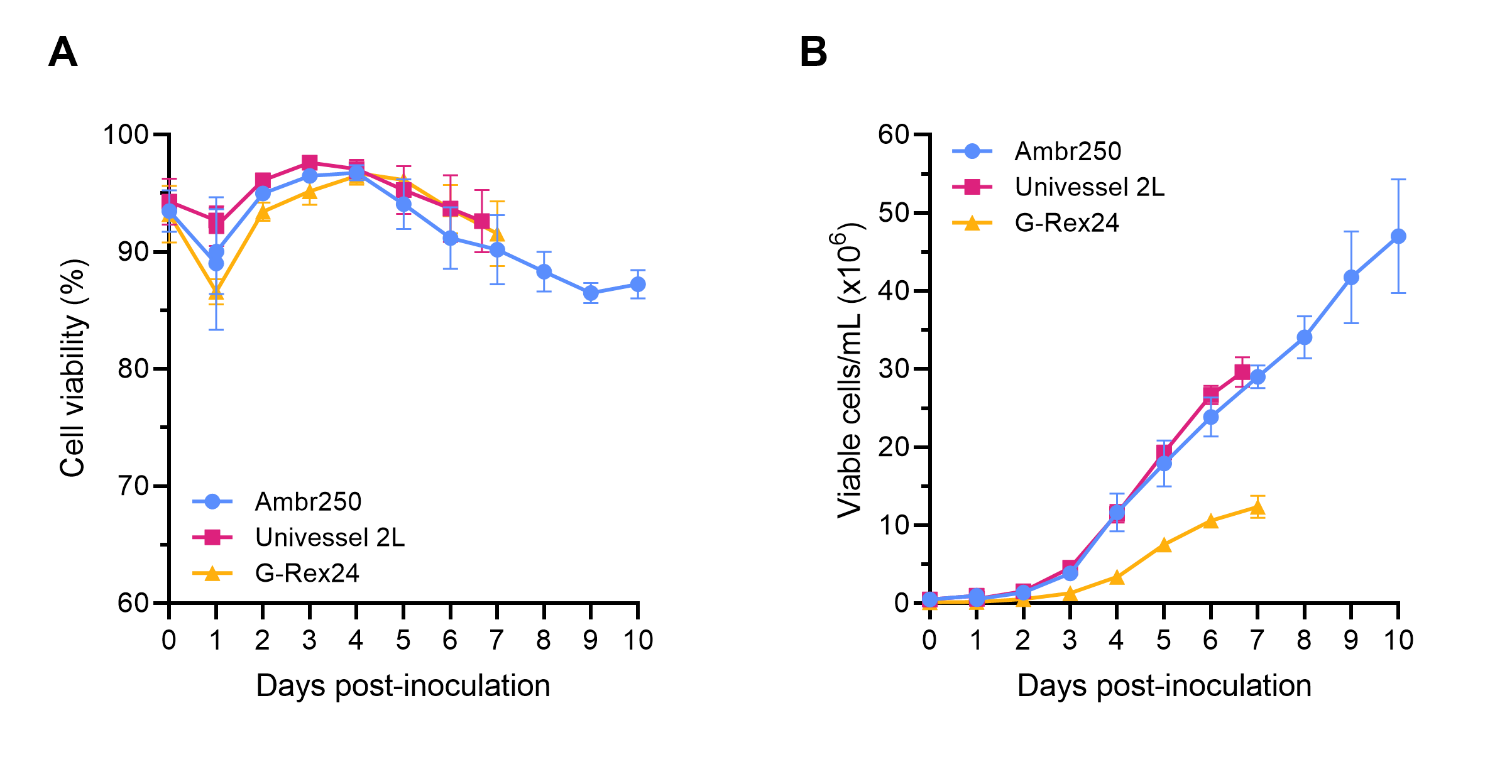
 **Figure S3: Extended CAR-T cell expansions in the 250mL perfusion stirred-tank bioreactor.**Anti-CD19 CAR-T cell expansions wee extended in the Ambr^®^ 250 from Day 7 to 10. **a)** Daily cell viabilities and **b)** daily viable cell densities. Data presented as mean ± SD of n=3 experimental replicates using one healthy donor with well plate data presented as the mean of n=3 technical replicate wells per experimental replicate (n=9).


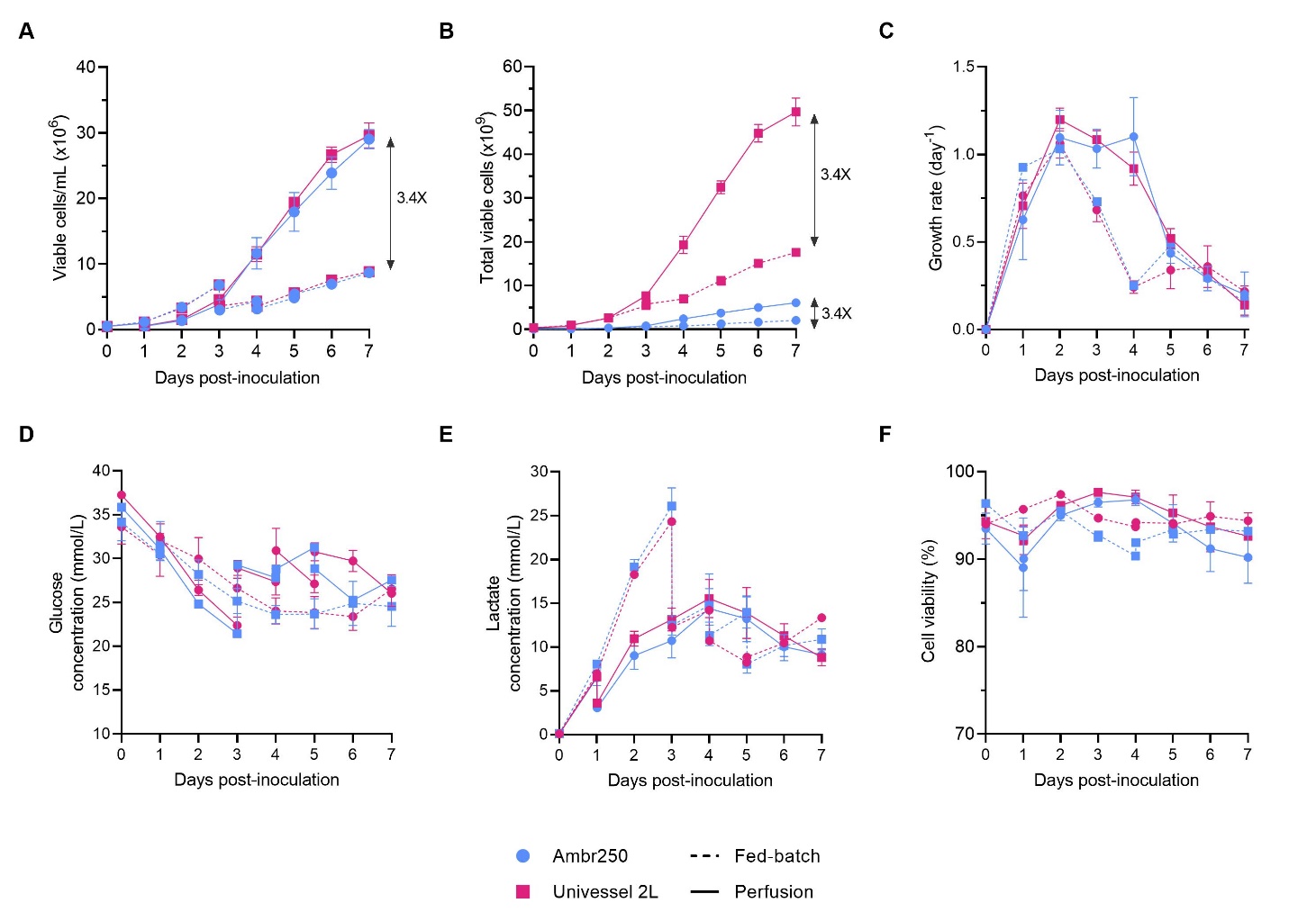
 **Figure S4: Comparison of expansion of T cells in fed-batch versus CAR-T cells perfusion in 250mL and 2 L stirred-tank bioreactors.**Cells were inoculated in the Ambr^®^ 250 and Univessel^®^ 2 L stirred-tank bioreactors expanded for seven days in fed batch (T cells) and perfusion mode (CAR-T cells). In fed-batch, the bioreactor working volume was increased by 100% on Day 3 and by 25% on Day 4, followed by a 40% manual medium exchange on Day 5. In perfusion, the bioreactor working volumes were increased by 110% on Day 1, and perfusion was initiated on Day 2 at 1.0 vessel volume exchanges per day until the end of experiments. **a)** Daily viable cell concentrations, **b)** total viable cells, **c)** cellular growth rates, **d)** glucose concentrations, **e)** lactate concentrations and **f)** cell viabilities. Perfusion data presented as mean ± SD of n=3 experimental replicates and fed-batch data presented as n=1 experimental replicate using one healthy donor.


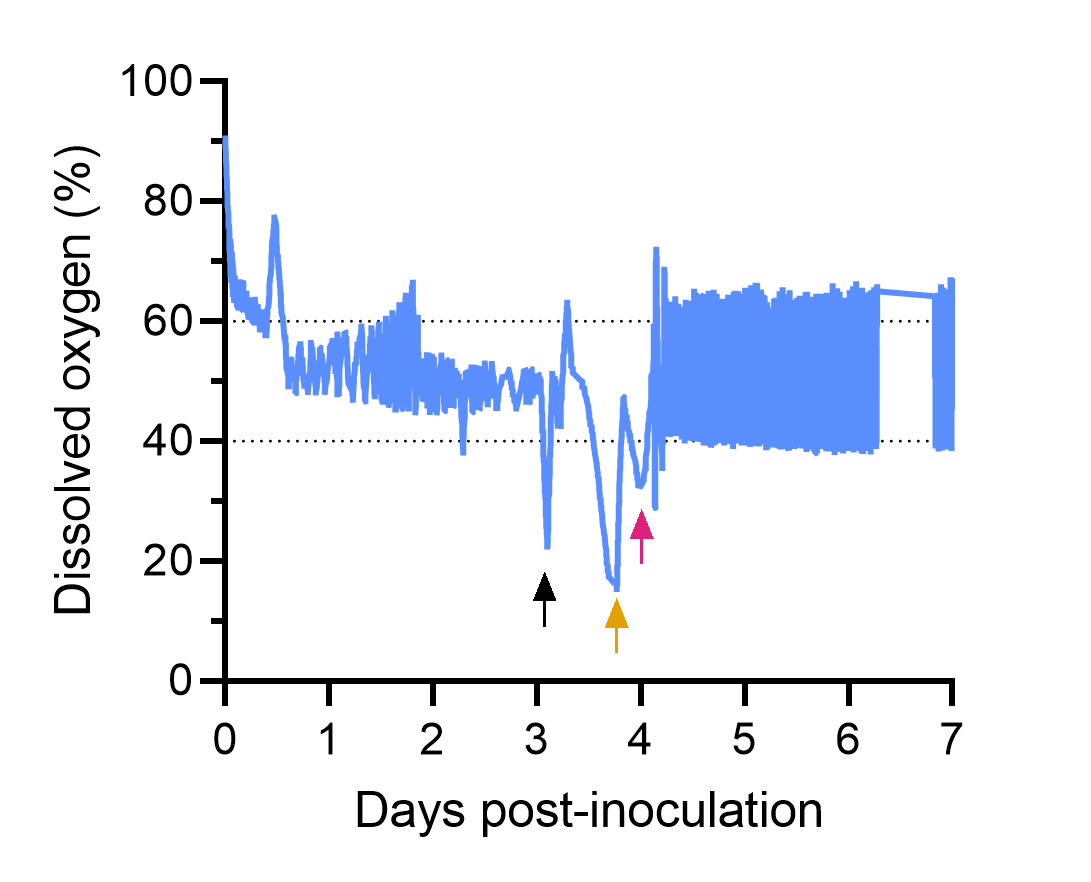
 **Figure S5: Headspace gassing alone was insufficient to maintain dissolved oxygen setpoint in preliminary 2 L stirred-tank bioreactor experiments.**Dissolved oxygen data are shown from a preliminary 2 L stirred-tank bioreactor perfusion run using non-modified T cells. By Day 3, the original headspace-only gassing strategy could no longer maintain the target dissolved oxygen setpoint of 50 ± 10%, so the headspace oxygen flow was increased to its maximum rate (black arrow). By Day 3.75, the increased headspace gassing was insufficient, and an initial sparging strategy was added (yellow arrow). On Day 4, the sparging rate was further increased (purple arrow), which successfully maintained dissolved oxygen within the desired range until the end of culture.


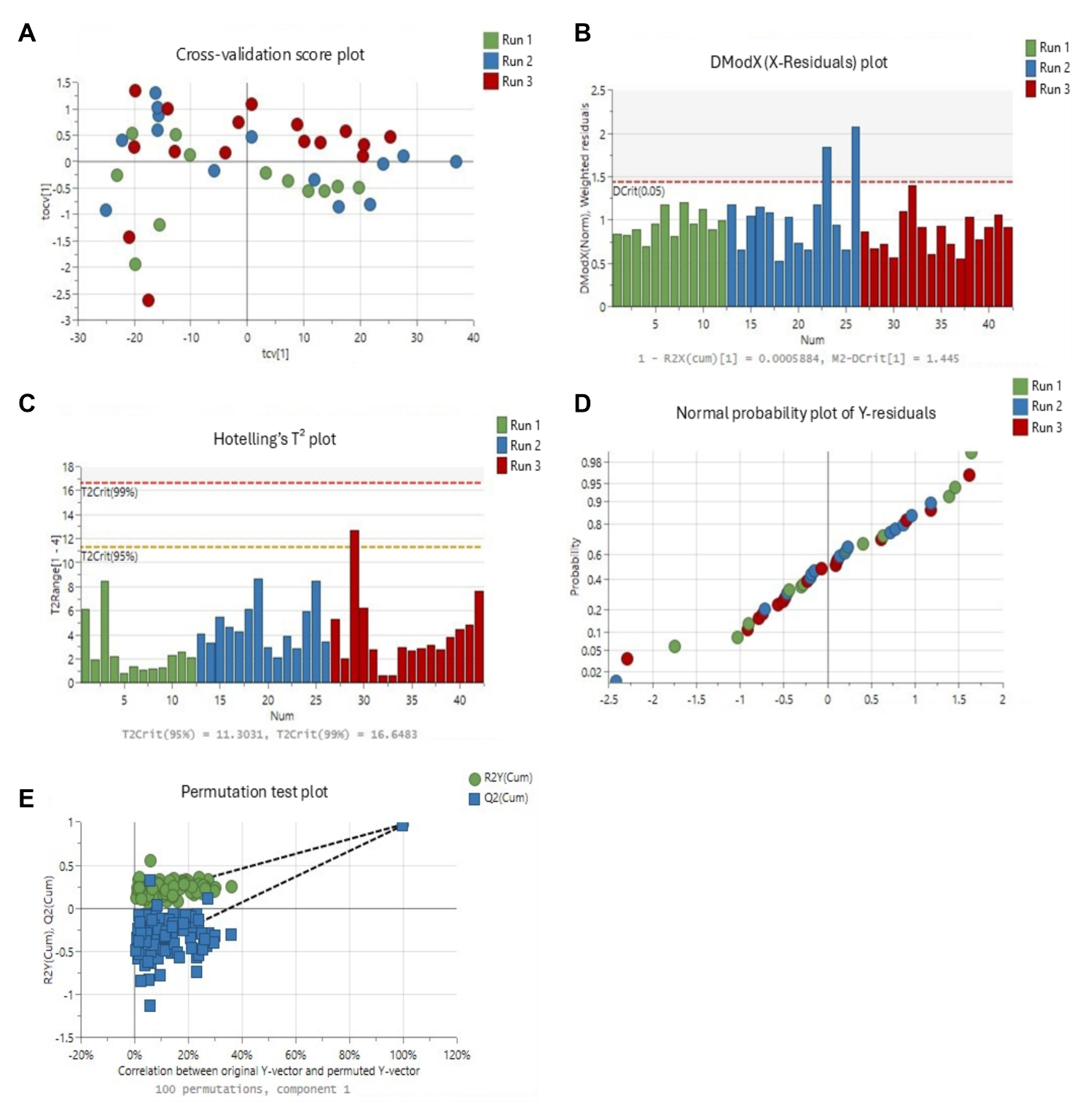
**Figure S6: OPLS model validation diagnostics for capacitance-based prediction of viable cell densities across three 2 L stirred-tank bioreactor CAR-T cell cultures.
a)** Cross-validated score plot showing predictive and orthogonal scores for Runs 1–3; the overlap of scores indicates consistent internal predictive behaviour. **b)** DModX (X-residuals) plot showing that most samples fall below the 95% confidence limit (Dcrit), with only isolated moderately elevated residuals. **c)** Hotelling’s T² plot indicating that most samples fall below the 95% and 99% confidence limits (T²Crit), with only a few isolated high-leverage samples and no systematic outlier behaviour. **d)** Normal probability plot of Y-residuals showing approximate normality and absence of systematic prediction bias across all runs. **e)** Permutation test (100 permutations) demonstrating a negative Q² intercept and permuted Q² values substantially lower than the original model, confirming that the model’s predictive structure is stronger than expected by chance.


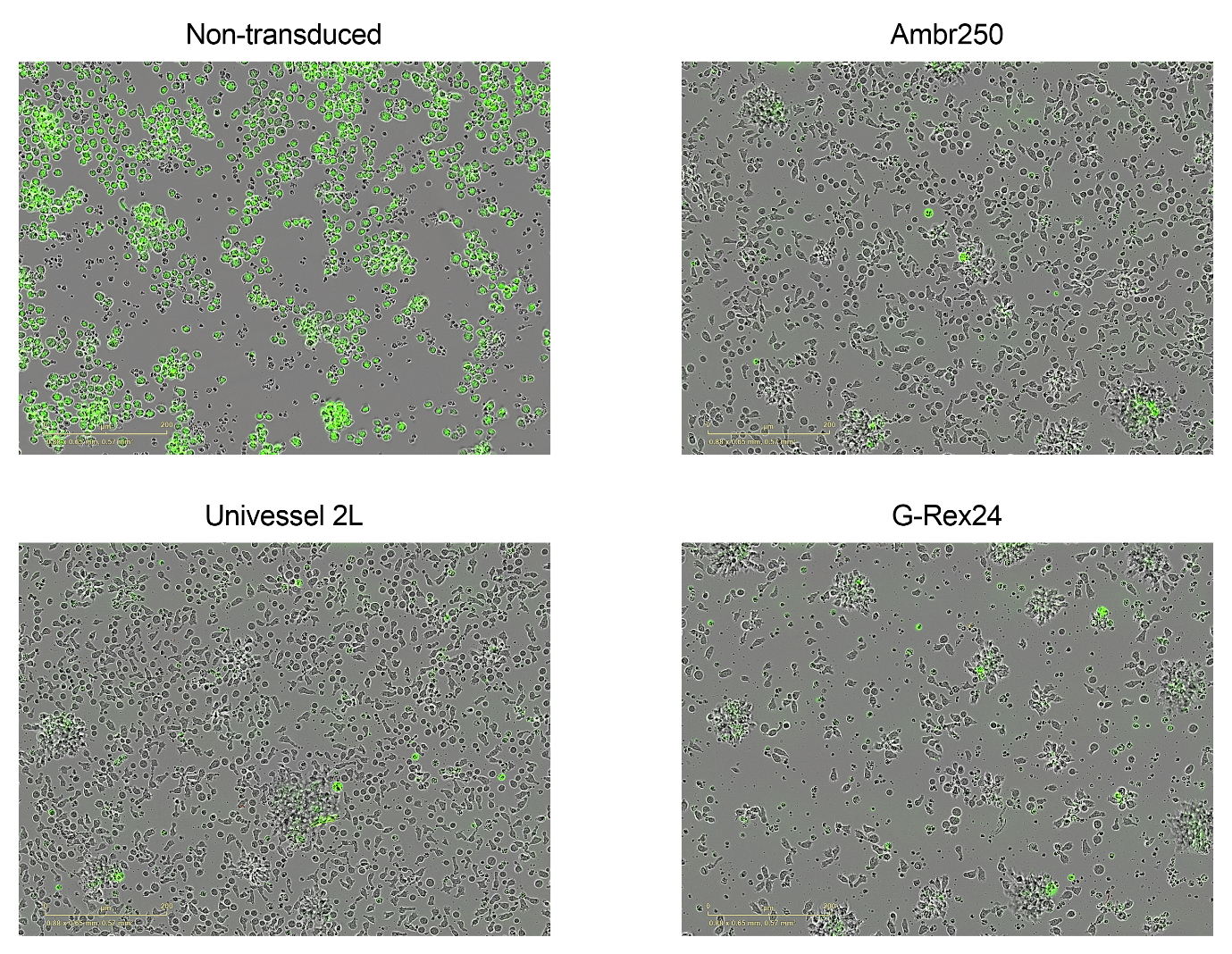


**Figure S7: Representative 20X microscopy images from anti-cancer CAR-T cell cytotoxicity assay.**Harvested CAR-T cell cytotoxicity was assessed in vitro via fluorescent microscopy by 1:1 co-culture with Nuclight Green NALM6 target cells. Images taken at 72 hours.
